# Tendinopathy: The Interplay between Mechanical Stress, Inflammation, and Vascularity

**DOI:** 10.1002/advs.202506440

**Published:** 2025-08-26

**Authors:** Renate Gehwolf, Herbert Tempfer, Nevra Pelin Cesur, Andrea Wagner, Andreas Traweger, Christine Lehner

**Affiliations:** ^1^ Institute of Tendon and Bone Regeneration Paracelsus Medical University Salzburg 5020 Austria; ^2^ Austrian Cluster for Tissue Regeneration Vienna Austria

**Keywords:** angiogenesis, inflammation, mechanical load, metabolism, tendinopathy

## Abstract

Tendinopathy is a long‐lasting, debilitating disease that not only affects patients' individual lives, but also imposes a significant socioeconomic burden. Due to an insufficient understanding of the underlying pathomechanisms, treatment options remain limited. Although tendinopathy is recognized as a multifactorial disorder, known risk factors and predisposing conditions have largely been studied in isolation. In this review, it is aimed to examine the various pathomechanisms identified to date and synthesize them into a unified concept. Particular emphasis is placed on key drivers believed to be critical in the onset and progression of the disease—namely mechanical stress, inflammation, and vascular changes. By exploring molecular signaling pathways and conditions involved in tendinopathy, how these mechanisms interact and reinforce one another is illustrated, forming a complex and dynamic signaling network, referred to as the tendinopathic loop. In the future, this network is expected to become even more intricate with the inclusion of emerging concepts such as intratendinous pressure and the potential influence of metabolic factors.

## Graphical abstract

This review explores the complex and self‐reinforcing cycle of mechanical stress, inflammation, and altered vascularity in tendinopathy—a common, painful tendon disorder. It integrates current findings on cellular, molecular, and biomechanical mechanisms, highlighting how mechanical overload, inflammatory signals, and abnormal blood vessel behavior contribute to disease progression. It also discusses emerging concepts like intratendinous pressure and metabolic influences.

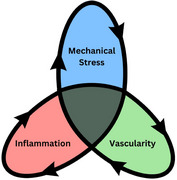

## 1 Introduction

The term “Tendinopathy” encompasses a broad range of tendon disorders that share common features but arise from different causes. This review aims to explore the primary mechanisms currently suspected to contribute to the development of this pathology. To date, our knowledge of the cellular and molecular processes behind the histopathological changes in tendon tissue, which lead to biomechanical deficits and pain, remains incomplete and fragmentary. It is widely acknowledged that tendinopathy is a complex, multifactorial disease, leading to diverse and sometimes conflicting theories about its onset and progression. This article seeks to consolidate and integrate the various pathophysiological mechanisms being discussed in the scientific community, with a particular focus on the interplay of mechanical stress, inflammation, and vascular changes, which are considered the key drivers of tendinopathy. With this approach we hope to introduce a new concept which may aid researchers and clinicians to tailor adequate future treatment options.

### 1.1 Epidemiology and Societal Impact

Epidemiological data from different countries show that the number of persons suffering from tendinopathy has been rising continuously over the last decades, affecting both professional and amateur athletes as well as people across all ages, this increase partly being due to an increasingly elderly and more active population.^[^ ^1^ ^]^ An EU report on the development of occupational diseases for selected musculoskeletal disorders corroborates these data demonstrating that the number of tenosynovitis and enthesopathies has been increasing by around 10% to 20% in the period from 2013 to 2019.

Since prevalence and incidence rates differ between studies due to differences in patient cohorts (athletes, workers, general population), study design, tendon type affected, it is impossible to cite a meaningful number valid for all. For a more comprehensive overview of the prevalence of various types of tendinopathy, interested readers may refer to two recent systematic reviews including in total 44 studies and an extensive review by Hopkins et al. (2016), which concludes that sex does not appear to be a significant factor in tendinopathy.^[^ ^2^, ^3^, ^4^ ^]^

In addition to their painful and debilitating impact on patients’ daily lives, tendon disorders represent a still underestimated socioeconomic burden.^[^ ^2^ ^]^ For the Netherlands alone the absolute annual costs of Achilles tendinopathy have been estimated at more than 21 million euros.^[^ ^5^ ^]^

## 2 Tendinopathy

### 2.1 Definition

The terminology surrounding tendon pathology has evolved alongside advancements in our understanding of its underlying mechanisms. Initially, the term “tendinitis” was used, based on the assumption that inflammation played a central role. However, as subsequent studies failed to detect immune cells in affected tendons, the condition was reclassified as degenerative rather than inflammatory, leading to the adoption of the term “tendinosis.” More recently, advanced detection methods have identified immune cells and inflammatory mediators in early‐stage tendinopathy, suggesting that inflammation may indeed play a role in the disease process. As a result, the broader and more neutral term “tendinopathy” is now preferred in reference to its etiology.

### 2.2 Clinical and Histological Features of Tendinopathies

When a patient reports a restricted range of motion in a limb, pain upon palpation, and symptoms such as redness, tenderness, and swelling, a physician will likely suspect tendinopathy. In most cases, ultrasound and X‐ray imaging provide additional insights, not only confirming the presence of blood vessels and calcific deposits but also identifying their precise location.^[^ ^6^ ^]^

Histological analysis reveals distinct characteristics in diseased tendons. Key features are disorganized collagen fibers and extracellular matrix degradation. Some tendons exhibit fatty infiltrations, and hypercellular regions, often accompanied by an increased presence of blood vessels, innervation, and immune cells. In contrast, others primarily display calcifications within a largely avascular matrix. This variability in structural changes likely reflects the diverse underlying pathomechanisms, highlighting the multifactorial nature of the disease.

Interestingly, tendinopathic tendons are not always associated with pain. Many patients experiencing pain show no significant histological changes in their tendons, whereas highly degenerated tendons are often asymptomatic. Overall, the exact origin of pain in tendinopathy remains largely unknown. Current research suggests that nerve ingrowth and the release of neuronal mediators, such as glutamate and substance P, play a central role in pain development.^[^ ^7^, ^8^ ^]^

**Figure 1:**
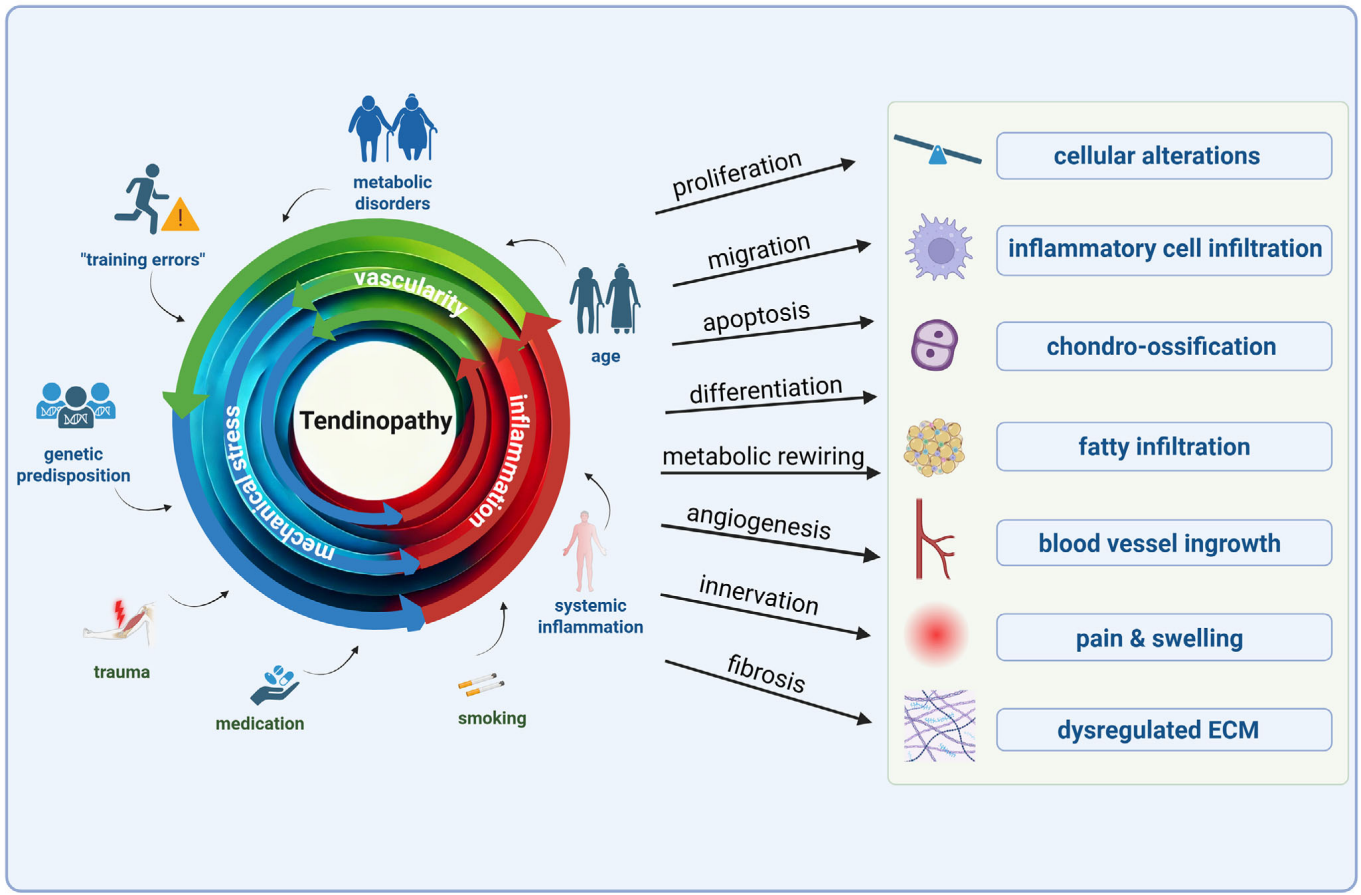
Overview of the conditions predisposing individuals to the development of tendon disease: In addition to the three main drivers—mechanical stress, inflammation, and vascularity—a variety of extrinsic and intrinsic factors feed into the "tumble wheel” of tendinopathy.” These combined influences ultimately lead to the pathological alterations outlined in the right column. Created with BioRender.com.

Generally, the conditions leading to signs of tendinopathy are manifold, comprising intrinsic as well as extrinsic risk factors summarized in **Figure** **1**. These factors induce a variety of different processes such as proliferation, apoptosis and fibrosis leading to alterations in the tissue. Metabolic rewiring is increasingly recognized as a central contributor to the pathophysiology of tendinopathy. It is characterized by a general shift from oxidative phosphorylation toward glycolysis, even under normoxic conditions. This metabolic shift causes maladaptive cell behavior and matrix degradation. It is characterized by an increased lactate production,^[^ ^9^ ^]^ an altered redox balance, and local acidosis, which may exacerbate tendon matrix degradation and chronic inflammation.^[^ ^10^ ^]^ Moreover, tenocytes in a tendinopathic tissue exhibit mitochondrial dysfunction and an upregulation of glycolytic enzymes,^[^ ^11^, ^12^ ^]^ suggesting a maladaptive response to mechanical and inflammatory stress. Mitochondrial dysfunction can disrupt lipid metabolism, particularly fatty acid oxidation, resulting in intracellular lipid accumulation—a characteristic feature of chronic tendinopathy. ^[^ ^13^ ^]^

## 3 Tendon Tissue

### 3.1 Cellular Compartment

Until not so long ago, tendons were considered “practically dead” and to consist primarily of tightly packed collagen fibrils with relatively few cells arranged in a bead‐like fashion between these bundles and the interfascicular matrix.^[^ ^14^ ^]^ These cells were generally described as rather immature round, ovoid tenoblasts and spindle‐shaped mature tenocytes.^[^ ^15^ ^]^ Overall, they have been considered a relatively homogeneous cell population, distinguished from normal fibroblasts by tendon‐specific markers such as scleraxis, tenomodulin, and Mohwak. Only recently, with the advent of high‐throughput sequencing technologies allowing high‐resolution profiling it has become increasingly clear that the cells within the tendon are much more heterogeneous than previously assumed. In two studies, examining human tendon cells ex vivo and in vitro by using single cell multiomics, at least five different tendon cell populations were identified in human tendon samples ex vivo, some of which share important gene expression markers.^[^ ^16^, ^17^ ^]^ It is not yet clear whether these different cell groups are separate cell populations or whether they share common precursors. All of these tenocytes express COL1A1/2 and are further classified into subgroups based on their unique gene expression profiles. These subpopulations are associated with specific functional traits, including microfibril formation, proinflammatory activity, fibro‐adipogenic differentiation, chondrogenic potential, or smooth muscle‐like mesenchymal characteristics.^[^ ^16^ ^]^ Interestingly, these newly identified different cell populations are to be found in both healthy as well as diseased tendons, albeit composition and quantity differ considerably.

**Figure 2:**
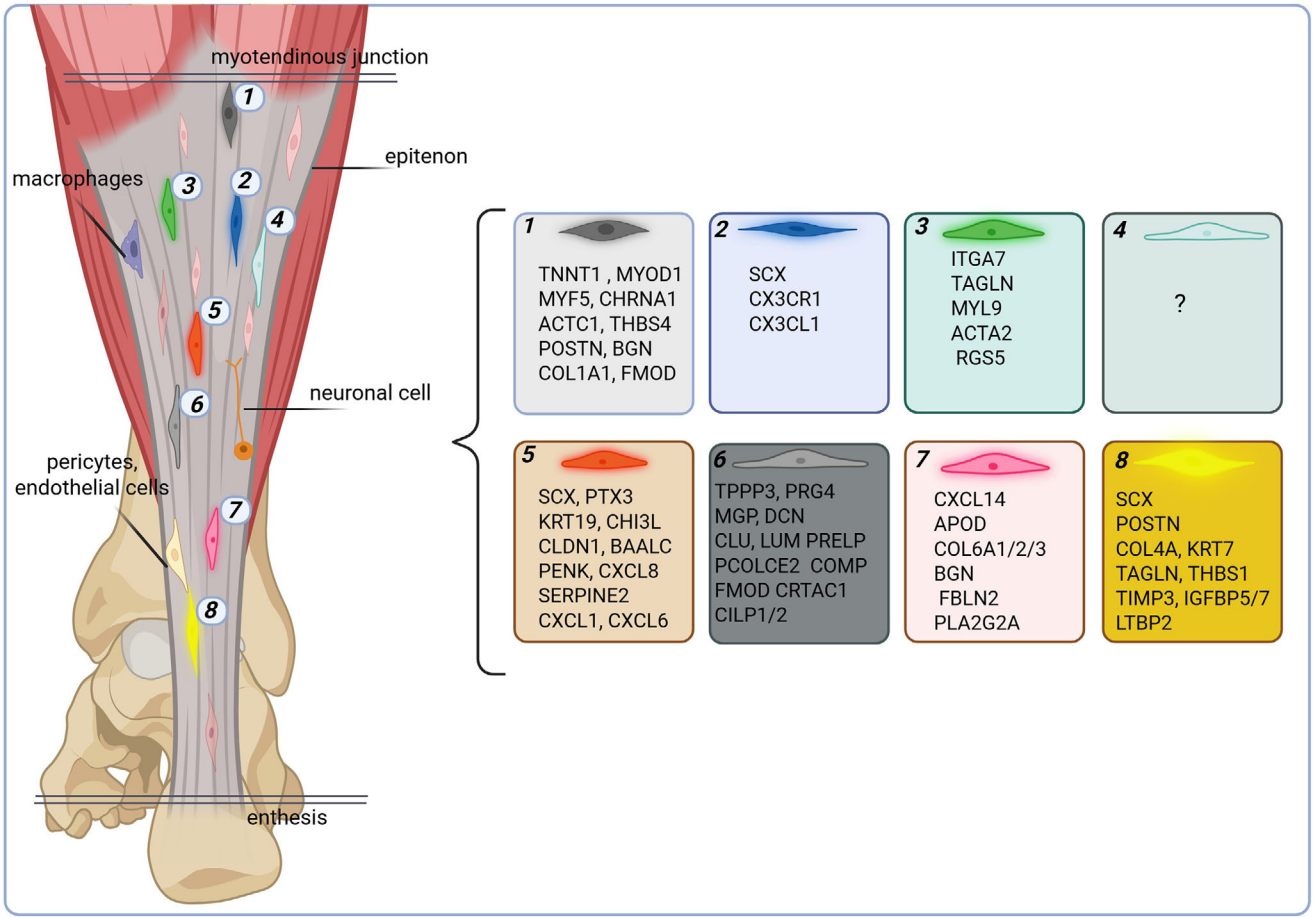
Illustration of the various cell populations identified within tendons. In addition to endothelial cells, macrophages, and neural cells, which can be readily distinguished by their characteristic marker profiles, several other tendon‐resident cells have been identified. These are considered distinct stromal and tenocyte subpopulations. Created with BioRender.com Adapted with permission.^[^ ^20^ ^]^ Copyright 2025, Elsevier.

Additionally, in a very recent study applying a single nucleus and spatial transcriptomic profiling approach 12 distinct cell types were found to be present in healthy human hamstring tendons confirming the presence of two (Mkx^+^ or Pdgfra^+^) fibroblast cell types. Next to these two distinguished fibroblasts subpopulations, endothelial cells, mural cells, immune cells, and in tendons previously unreported cell types, including different skeletal muscle cell types, satellite cells, adipocytes, and undefined nervous system cells were identified.^[^ ^18^ ^]^ These findings suggest the presence of multiple specialized tendon cell subtypes, which are likely to serve different functions, the contributions of which to the development of tendinopathy are only beginning to be unraveled (**Figure** **2**). Adipocytes were confirmed through gene expression (e.g., PLIN1, ADIPOQ) and spatial transcriptomics, which showed them to be localized near PDGFRA+ fibroblasts and skeletal muscle cells.^[^ ^18^ ^]^ Adipocytes play a complex role in tendinopathy, contributing to both tissue degeneration and impaired healing. In pathological tendon tissue, fatty deposits are often observed, particularly in chronic tendinopathy and post‐injury scenarios.

However, fatty infiltrations in tendinopathic tendon do not necessarily indicate erroneous differentiation toward adipocytes expressing canonical markers such as PPARG, Perilipin etc. In ageing tendon, lipid droplets are commonly found throughout the tendon, in a large variety of cells. A transgenic model of tendon ageing, the SPARC knockout mouse, shows a dramatic increase of lipid accumulation in tendon fibroblasts, suggesting a change of tendon cell phenotype rather than invasion of adipocytes or erroneous stem cell differentiation.^[^ ^19^ ^]^

The varying number of identified tendon subpopulations between different multiomic studies might also partly be due to whether whole cells or nuclei were used, to number of cells/nuclei included, and to different data analysis approaches, such as different parameter and threshold settings. Moreover, it has to be kept in mind that whole tendon samples were digested and subsequently subjected to single cell or single nuclei RNA seq analysis. So, the cell populations defined have been derived from a cell pool containing cells not only from the tendon proper, but also from the sheath compartment (epitenon/endotenon) and possibly also from the surrounding fatty tissue in case it had not been removed before the tissue digestion step. This is important to take into account, since so far, markers defining subpopulations have only in part been allocated to distinct regions of the tendon by using immunohistochemistry or spatial transcriptomics. TPPP3, a protein known to induce tubulin polymerization, for instance unequivocally has been shown to only localize to cells within the epitenon and paratenon of healthy tendons and is gaining emerging interest as it seems to play a role in adult and neonatal tendon regeneration.^[^ ^16^, ^21^, ^22^ ^]^ Also, a specific muscle‐tendon progenitor (MTP) subpopulation present at human myotendinous junctions (MTJ) has only recently been discovered at single‐cell resolution. This MTP population appears to have a stem cell character, as it simultaneously expresses muscle and tendon marker genes, which endows these cells with a certain differentiation potential.^[^ ^23^ ^]^ That the MTJ represents a specialized compartment with expression of specific proteins including COL22, tetraspanin‐24 (CD151), and the cartilage intermediate layer protein 1 (CILP1) has also been shown by others.^[^ ^24^, ^25^, ^26^ ^]^ A proteomic analysis of this region in mice, based on over 3,000 high‐resolution spatial proteomic profiles, revealed enhanced network connectivity, with 206 proteins exhibiting a local abundance maximum in this zone.^[^ ^24^ ^]^

**Figure 3:**
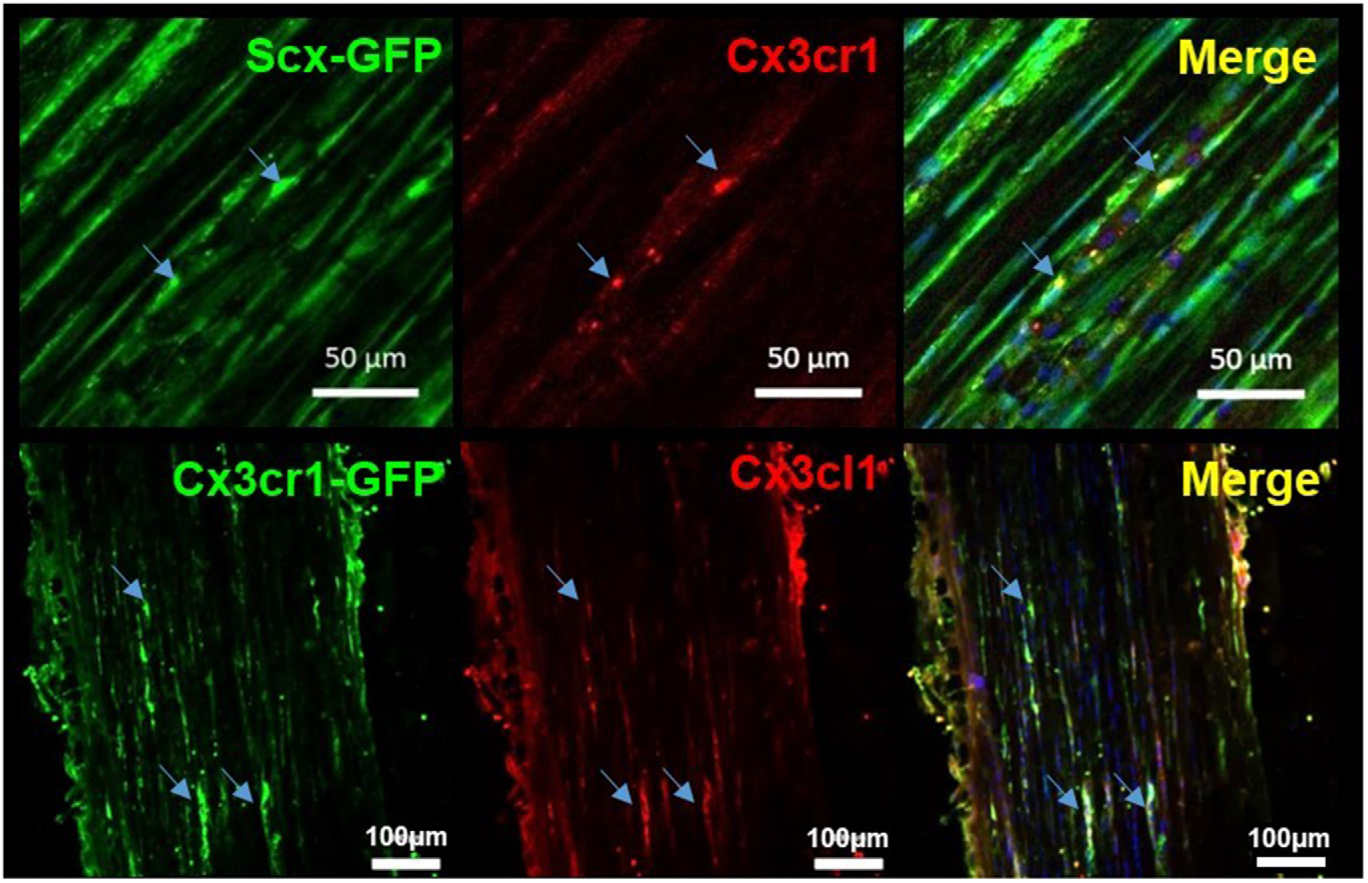
Tenophages: There is a tendon resident cell type co‐expressing both tendon‐ and macrophage associated markers. Expression of CX3CL1 and CX3CR1 in the tendons of Scx‐GFP and Cx3cr1‐GFP mice is shown on cryosections of Achilles tendons from transgenic Scx‐GFP A) and Cx3cr1‐GFP B). Arrows indicate cells that co‐express the respective proteins. Reproduced under the terms of the CC‐BY license.^[^ ^29^ ^]^ Copyright 2019, The Company of Biologists.

Although often considered immune‐privileged, healthy tendons also contain immune cells. So have T cells, albeit present in only low numbers, been detected in close proximity to tenocytes and mast cells adjacent to blood vessels in the interfascicular matrix and the epitenon, a connective tissue sheath surrounding the tendon.^[^ ^27^, ^28^ ^]^ Interestingly, a novel immune‐relevant cell population termed “tenophages” was recently identified. These cells exhibit both tendon‐associated markers, such as Scx, and macrophage‐associated markers, including CD68, CD163, and F4/80. Additionally, tenophages express both the ligand and receptor of the CX3CL1/CX3CR1 axis, a signaling pathway known to facilitate communication between tissue‐resident cells (e.g., neurons) and the immune system (e.g., microglia) in other tissues, like, e.g., the brain (**Figure** **3**).

In tendons, these cells are likely to serve a surveillance function, monitoring tissue integrity. When tissue damage surpasses their capacity to clear cellular debris, they may recruit immune cells from the circulation by upregulating fractalkine, thereby contributing to the inflammatory response.^[^ ^29^ ^]^

In the light of the studies mentioned and all the future studies to come, a consensus statement on which markers should be implemented to distinguish different cell clusters and fibroblast subtypes would greatly facilitate inter‐study comparability of data.

### 3.2 The Extracellular Matrix

The extracellular matrix (ECM) is fundamental in the formation and maintenance of soft connective tissues, such as tendons. Resident cells within the tissues are responsible for ECM synthesis during development, its sustainment throughout life, its degradation following disease or injury, and its remodeling during the repair process. In turn, the ECM influences cellular functions, including cell migration, cell proliferation, cell differentiation, and survival. These cell‐ECM interactions are governed not only by the biochemical composition of the ECM, but also by its mechanical properties, which enable cells to sense matrix characteristics.^[^ ^30^ ^]^ This is the basis to control matrix homeostasis, ensuring the preservation of structural integrity and functionality.

Tendons are subjected to some of the highest mechanical demands in the body. Their ability to withstand these high loads is attributed to the specialized structural organization and composition of the ECM. Although the tendon ECM comprises numerous proteins, glycoproteins, and small leucine‐rich proteoglycans (SLRPs), its mechanical properties primarily depend on collagen fibrils, elastic fibers, glycosaminoglycans (GAGs).^[^ ^31^, ^32^, ^33^, ^34^ ^]^ Besides these components, numerous matricellular proteins (MPs) are found in the tendon ECM, being a diverse family of noncollagenous ECM proteins that also appear in body fluids. While collagens, SLRPs, and GAGs provide structural support to cells and the ECM, MPs do not have a primarily structural function but can modulate cell‐matrix adhesion, migration, proliferation, and differentiation.^[^ ^35^ ^]^ They also induce the expression of proteolytic enzymes and, upon undergoing proteolysis themselves, release biologically active fragments that trigger signaling processes involved in wound healing and fibrosis. The best‐described MPs in tendons include thrombospondins (THBSs), tenascins (TNs), periostin (POSTN), cellular communication network (CCN) family members (e.g., CCN2/CTGF), and Secreted Protein Acidic and Rich in Cysteine (SPARC), also referred to as osteonectin.^[^ ^36^ ^]^ THBS‐5, also known as cartilage oligomeric matrix protein (COMP), tenascin C and periostin, a protein that interacts with tenascin C, have been reported to be upregulated in mechanically loaded tendons,^[^ ^37^, ^38^, ^39^ ^]^ whereas CCN_2_ plays dual roles in tendon pathology and repair with expression levels ranging from being increased, reduced, or comparable to those in healthy tissues.^[^ ^31^, ^40^ ^]^ A matricellular protein emerging to be relevant in the context of mechanotransduction is SPARC. It influences cell attachment, morphology, motility, proliferation, growth factor signaling, and ECM dynamics.^[^ ^41^ ^]^ Mice lacking SPARC exhibit notably thinner Achilles tendons, increased expression of adipogenic marker genes, and significant intracellular lipid accumulation.^[^ ^19^ ^]^

#### 3.2.1 The Dysfunctional ECM

**Figure 4:**
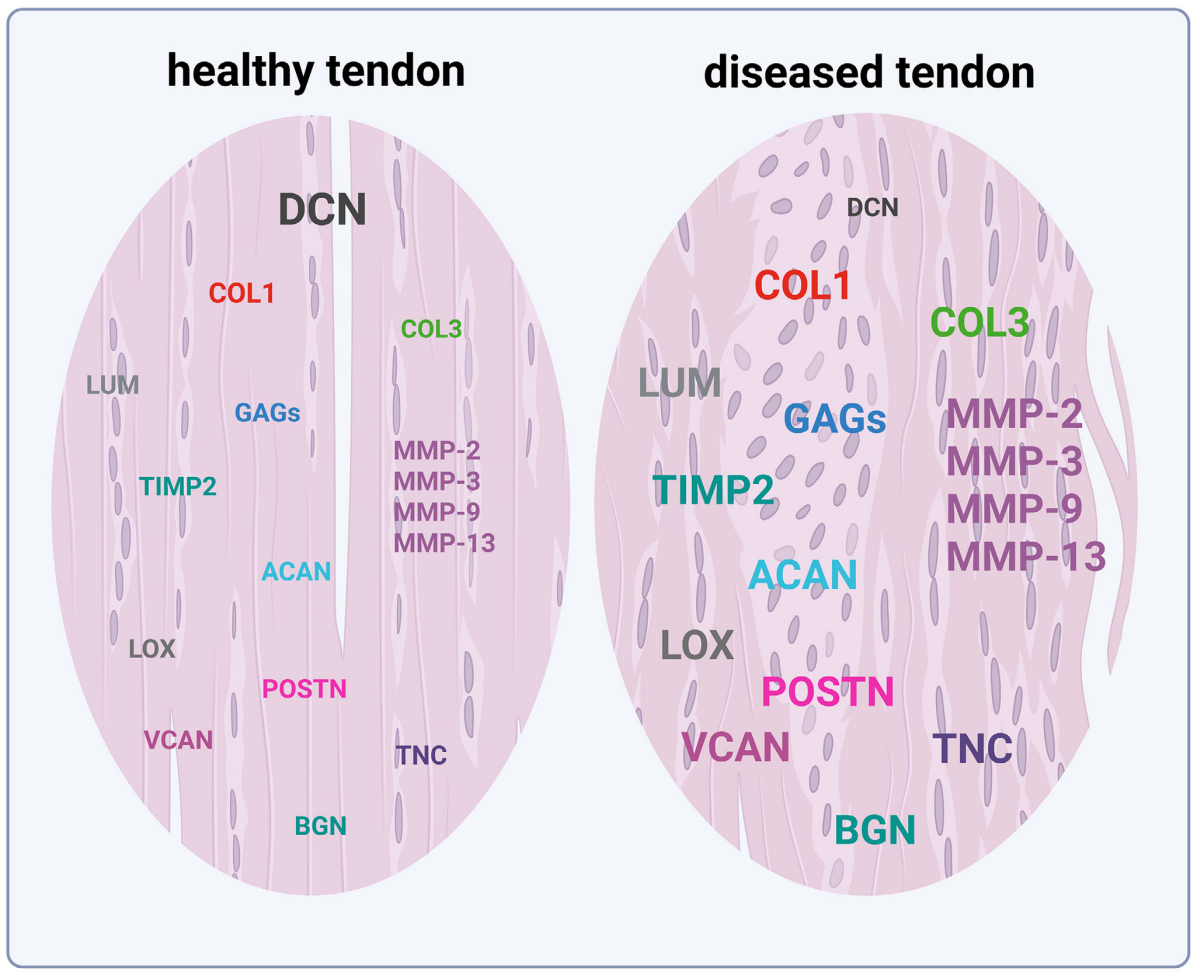
Pathologic shift in ECM: This scheme shows the shift in abundances of selected ECM proteins changed in tendinopathic tendons. Up‐ and downregulation of matrix components, respectively, is represented by font size with smaller letters indicating lower and larger letters indicating increased expression. Created with BioRender.com.

The maintenance of structural, functional, and compositional properties in tendons is strongly influenced by mechanical loading and the resulting downstream biochemical signals.^[^ ^42^ ^]^ Aberrant mechanical loading, whether excessive or insufficient, disrupts this balance, leading to secretion of inflammatory cytokines and chemokines, and, matrix dysregulation.^[^ ^43^ ^]^ This dysregulation often manifests as the overproduction of matrix‐degrading enzymes and shifts in the synthesis of matrix components, as visualized in **Figure** **4** and summarized in **Table** **1**.^[^ ^44^ ^]^ Whereas Table 1 provides information on altered ECM protein abundances under pathological conditions, **Table** **2** outlines the potential effects of these changes on pathomechanisms driving the disease.

**Table 1:** Displays ECM proteins described to be differentially regulated in tendinopathic tissues.

ECM component	Abbreviation	Function	Species	Expression in disease	Reference
Aggrecan	ACAN	proteoglycan water‐binding capacity	human	mRNA up in painful tendinopathy	[45]
			ovine	mRNA increased in overstressed tensile tendon; decreased in stress‐deprived tensile tendon	[46]
			ovine	decreased mRNA level in flexor tendinopathy	[47]
Biglycan	BGN	a small leucine‐rich repeat proteoglycan (SLRP)	human	mRNA expression up in painful tendinopathy	[45]
			ovine	mRNA increased in overstressed tensile tendon	[46]
Collagen 1	COL1	fibrillar collagen structural protein	human	increased in human Achilles tendinopathy	[48]
			ovine	decreased mRNA level in flexor tendinopathy	[47]
			ovine	increased in overstressed and stress‐deprived tensile tendon	[46]
Collagen 3	COL3	fibrillar collagen structural protein	human	increased in human Achilles tendinopathy	[48]
			ovine	increased mRNA level in flexor tendinopathy	[47]
			ovine	increased in stress‐deprived tensile tendon	[46]
Decorin	DCN	a small leucine‐rich repeat proteoglycan (SLRP)	ovine	decreased in overstressed and stress‐deprived tensile tendon	[46]
			ovine	decreased mRNA level in flexor tendinopathy	[47]
Keratan sulfate	KS C4S C6S	major glycosaminoglycans	ovine	upregulated in overstressed and stress‐deprived tensile tendon	[46]
Chondroitin 4‐sulfate			ovine	upregulated in overstressed and stress‐deprived tensile tendon	[46]
Chondroitin 6‐sulfate			ovine	upregulated in overstressed and stress‐deprived tensile tendon	[46]
Lumican	LUM	small leucine‐rich proteoglycans (SLRP) regulates collagenous matrix assembly	human	increased in human Achilles tendinopathy	[48]
			ovine	increased in overstressed and stress‐deprived tensile tendon	[46]
			ovine	increased mRNA level in flexor tendinopathy	[47]
Lysyloxidase	LOX	a copper‐containing amine oxidase facilitates covalent cross‐linking of both collagen and elastin fibers in connective tissue	ovine	increased levels in overstressed and stress‐deprived tensile tendon	[46]
			human	gene upregulated in tendinopathy	[49]
Matrix metalloproteinases	MMPs (MMP2, MMP3, MMP9, MMP13)	calcium‐dependent zinc‐containing endopeptidases; degrade ECM proteins and glycoproteins	human	increased expression of MMP2 and MMP9 in the diseased part of tendinopathic tendons	[48]
			rat	stress deprivation led to elevated expression of MMP‐13, and MMP‐3 in Achilles and supraspinatus tendon	[50]
Periostin	POSTN	a matricellular, nonstructural ECM protein; plays key roles in fibrillogenesis and cell migration	ovine	increased levels in overstressed and stress‐deprived tensile tendon	[46]
Tenascin‐C	TNC	large hexameric matricellular glycoprotein	ovine	increased mRNA level in flexor tendinopathy	[47]
			human	increased in human Achilles tendinopathy	[48]
Tissue inhibitors of metalloproteinase 2	TIMP2	Glycoproteins; key regulators of the metalloproteinases	human	increased expression of TIMP2 in the diseased part of tendinopathic tendons	[48]
			rat	stress deprivation led to elevated expression of Timp2 in Achilles and supraspinatus tendon	[50]
Versican	VCAN	large chondroitin sulphate proteoglycans	ovine	upregulated in stress‐deprived tensile tendon	[46]

**Table 2:** Overview of ECM proteins and their role in tendinopathy with special focus on their effects on inflammation, angiogenesis, and fibrosis.

ECM component	Role in tendinopathy	Effect on inflammation	Effect on angiogenesis	Effect on fibrosis
**Collagen type 1**	overproduced but disorganized in tendinopathy	no direct effect	indirect (structural support for vessels)	overproduced in fibrosis
**Collagen type 3**	upregulated during early repair & chronic degeneration	no direct effect	may facilitate neovessel formation via loose matrix	marker of early fibrosis; replaces collagen 1 in disorganized tissue
**MMPs**	ECM degradation &remodelling	pro‐inflammatory (MMP‐3 and‐9)	release of ECM bound VEGF	imbalance→3ECM degradation or fibrosis
**TIMPs**	inhibit MMPs and thus ECM breakdown and remodelling	anti‐inflammatory, but context‐dependent	may stabilize vessel ECM	over‐expression can promote fibrosis
**GAGs**	ECM matrix swelling and disrupt collagen breakdown	bind cytokines, modulate responses	bind VEGF and other pro‐angiogenic factors	disrupts ECM integrity and function
**ACAN**	accumulates in diseased tendons, ECM swelling	fragments may promote inflammation	no direct effect	ECM disorganization
**POSTN**	matrix stiffening, chronic degeneration	e. g., macrophage and NF‐kB activation	pro‐angiogenic	favors myofibroblast maintenance
**VCAN**	increases in early tendinopathy; cell migration	fragment acts as DAMP	enhances endothelial sprouting	disrupts collagen architecture
**TNC**	injury‐induced, elevated in degeneration areas	TLR‐4 activation	variable	regulates cell‐matrix interaction
**BGN**	collagen fibrillogenesis	TLR2/4 activator	limited effect	fibrotic enhancer
**DCN**	collagen alignment, TGF‐β regulator	anti/pro‐inflammatory depending on context	limited effect	key antifibrotic regulator
**LUM**	regulates collagen fibrillogenesis,	modulates innate immune responses	Indirect effect via ECM stiffness	promotes matrix stiffness in injury and fibrosis
**LOX**	matrix stiffening	indirect via stiff ECM guiding vessels	indirect via stiff ECM guiding vessels	crosslinks collagen → stiff, fibrotic matrix
**SPARC**	not yet fully described	indirect via e. g., immune cell‐ECM interaction	anti‐angiogenic	pro‐fibrotic

A significant driver of pathological changes of the ECM is the upregulation and hyperactivation of collagenolytic matrix metalloproteinases (MMPs), which, together with an imbalance of their inhibitors (tissue inhibitor of metalloproteinases or TIMPs), accelerate ECM turnover. Particularly, MMP‐2 and MMP‐9 are highly expressed and active in tendinopathic tissues, promoting excessive collagen degradation.^[^ ^51^ ^]^ The activation of pro‐MMP‐2 by plasmin further exacerbates matrix proteolysis, while proteases like cathepsins K and S degrade collagen fragments, contributing to tendon degeneration. Interestingly, both MMPs have been found to contribute to the breakdown of tight junction (TJ) proteins in endothelial cells, resulting in blood–brain barrier disruption and a corresponding increase in permeability.^[^ ^52^, ^53^, ^54^, ^55^ ^]^ Notably, these TJ proteins—occludin, claudin‐5, and ZO‐1—are also expressed in blood vessels within tendon tissue.^[^ ^56^ ^]^

In addition to the above‐mentioned enzymes, MMP‐3 and MMP‐13 are involved in the pathogenesis of tendinopathy being associated with excessive ECM turnover, which leads to structural and functional alterations.^[^ ^57^, ^58^ ^]^ The upregulation of MMP‐13 contributes to the transition from acute tendon injury to chronic tendinopathy by perpetuating collagen degradation and interfering with tissue repair processes. MMP‐3 and MMP‐9 are strongly up‐regulated in the early phase of tendinopathy promoting inflammation by degrading numerous matrix proteins and releasing danger associated molecular patterns (DAMPs). TIMPs, their specific inhibitors are usually anti‐inflammatory. However, an imbalance (both, too high or too low) can exacerbate inflammation. Both, MMP‐3 and MMP‐13 are upregulated by proinflammatory cytokines and mechanical stress, creating a feedback loop that exacerbates matrix breakdown in tendinopathic conditions.^[^ ^50^, ^59^ ^]^

Abnormal levels of GAGs and proteoglycans affect fiber sliding, matrix integrity, and the mechanical properties of tendons, such as increased stiffness, which raises the risk of tendinopathy and tendon tears. Table 1 summarizes ECM components described to be altered in tendinopathy. These changes impair the matrix's mechanical resilience and regenerative capacity.^[^ ^60^, ^61^ ^]^ Moreover, GAGs are directly involved in modulating local inflammation by binding cytokines and chemokines (e.g., IL‐1β, TNF‐α). As described for other musculoskeletal pathologies LUM can modulate innate immune responses via a direct interaction with macrophages.^[^ ^62^, ^63^, ^64^ ^]^ Fragments of ECM proteins like VCAN or ACAN are described as pro‐inflammatory mediators in various disease processes.^[^ ^65^, ^66^ ^]^ DCN is modulating inflammation via TGF‐β and Toll‐like receptors and its dysregulation can prolong inflammation as shown for lung fibrosis.^[^ ^67^ ^]^ These changes also create a microenvironment that favors pathological angiogenesis. MMP‐mediated ECM degradation exposes angiogenic factors like VEGF, while the disorganized ECM facilitates endothelial cell migration.^[^ ^68^ ^]^ This migration capacity is also influenced by GAGs, which bind and regulate proangiogenic factors creating gradients for endothelial cell migration.^[^ ^69^ ^]^ POSTN and VCAN have rather similar functions in angiogenesis favoring vessel sprouting.^[^ ^65^ ^]^

Overall, the increase in these and other ECM components in tendinopathy reflects a maladaptive repair response in the tendon tissue, often driven by mechanical overload, microtrauma, or chronic inflammation.

### 3.3 Vascular Bed

Early medical literature described tendons as being “virtually dead during life.” This conclusion was based on the observation that tendons are poorly vascularized and contain relatively few cells compared to other tissues. Although dye injection studies visualized the vascular and lymphatic networks of tendons, they were historically considered “nonviable cables”.^[^ ^70^ ^]^ A common physiological response to low tissue nutrition is the increased expression of proangiogenic factors, such as vascular endothelial growth factor (VEGF), which attract blood vessels. However, in healthy tendons, this response seems largely absent.^[^ ^70^ ^]^ Maintaining a state of hypo‐ or avascularity in tendons requires either the suppression of proangiogenic factors or the production of antiangiogenic factors. Both mechanisms have been described in certain areas, such as the hypovascular zones of sheathed tendons.^[^ ^71^ ^]^

**Figure 5:**
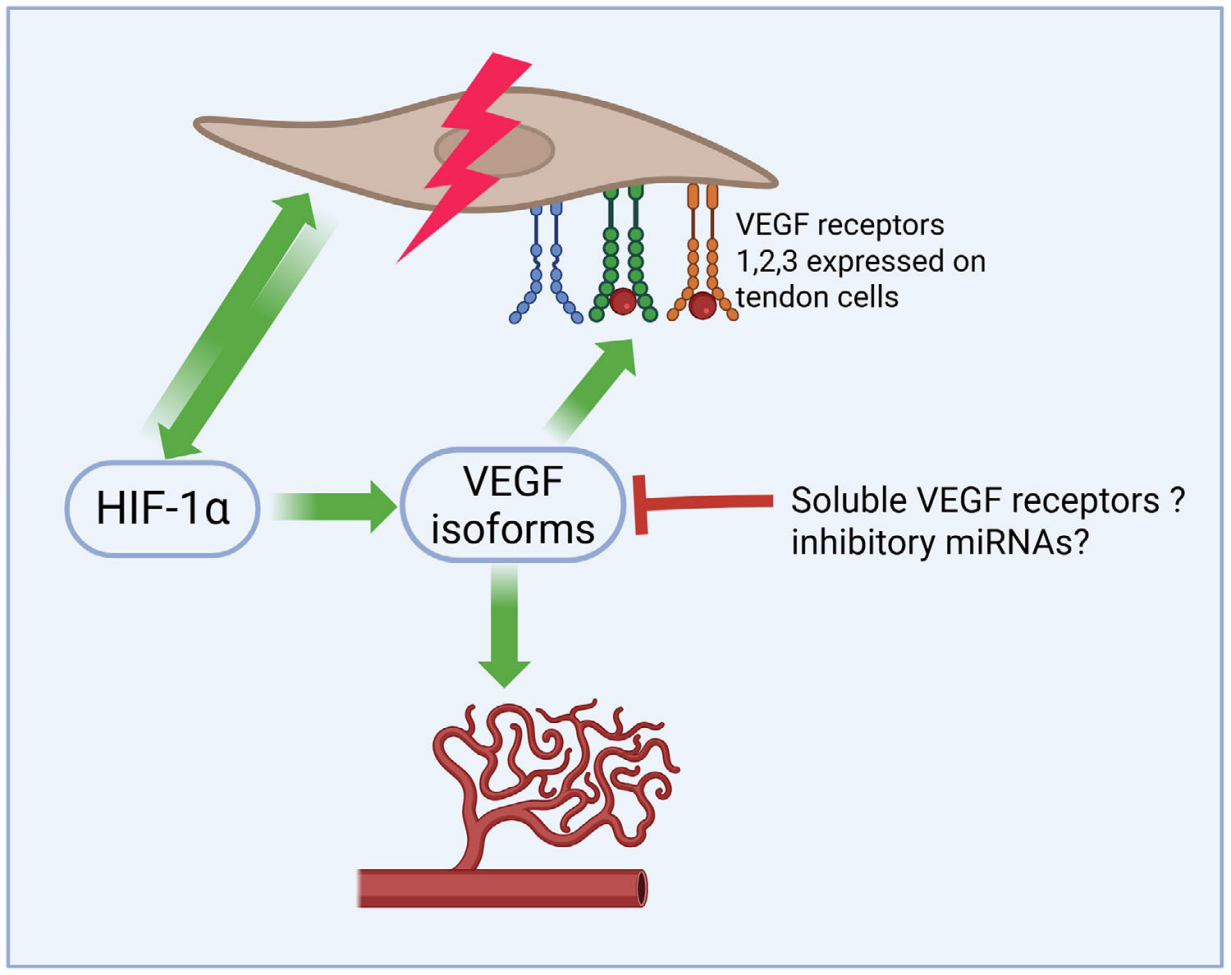
The three known isoforms of VEGF do not only promote blood‐ and lymph vessel growth, but also activate VEGF receptors expressed in tendon cells, leading to matrix disruption and the expression of inflammatory factors.^[^ ^72^ ^]^ HIF1, the well described major driver of hypoxia induced angiogenesis was recently found to exert direct, tendinopathy‐like degeneration in tendon cells, independent of VEGF‐signaling.^[^ ^73^ ^]^ Potential factors preventing healthy tendon from blood vessel ingrowth despite potentially low oxygen supply remain unknown. In other tissues like the cornea, scavenging soluble VEGF receptors are known to contribute to maintaining avascularity.^[^ ^74^, ^75^ ^]^ Created with BioRender.com.

Despite growing interest in the vascular mechanisms underlying tendon pathology, there remains a significant gap in our understanding of how these processes are regulated in healthy tendon tissue. In particular, the mechanisms that maintain the characteristic low vascularity of tendons under normal physiological conditions are poorly defined. It is reasonable to hypothesize that this avascular state is not merely a consequence of mechanical rigidity or structural density, but rather the result of a coordinated interplay of mechanical, molecular, and cellular factors. Mechanical stiffness and the dense extracellular matrix may physically limit vessel penetration; however, additional, yet unidentified biological mechanisms are likely involved. These may include the local expression of anti‐angiogenic factors, such as soluble vascular endothelial growth factor (VEGF) receptors that act as molecular decoys by binding and sequestering VEGF, thus preventing its pro‐angiogenic effects. Furthermore, microRNAs (miRNAs) could play a role by post‐transcriptionally regulating angiogenesis‐related genes—for example, by suppressing the translation of VEGF mRNA or other components of the angiogenic signaling cascade (**Figure** **5**).

The proangiogenic protein VEGF is found to be highly expressed in cells from fetal and injured human tendons, but its expression is significantly lower in intact adult tendons.^[^ ^76^ ^]^ This pattern is consistent with findings that VEGF is expressed in tissue samples from tendons that resemble an early stage of tendinopathy. In the same study, upregulation of markers associated with apoptosis and hypoxia was also observed. Notably, in vitro experiments using human tendon cells under normoxic and hypoxic conditions demonstrated stabilization of HIF1α under hypoxia. This stabilization was accompanied by a shift in the expression ratio of collagen types I and III, favoring type III, which suggests a fibrotic or degenerative response. Additionally, levels of proinflammatory cytokines such as IL6 and MCP‐1 were increased.^[^ ^77^ ^]^ Interestingly, recent work shows that HIF1α not only acts on tendon via the induction of angiogensis, but also exerts a direct effect on matrix degeneration through a maladaptive tissue response to chronic overload.^[^ ^73^ ^]^

**Figure 6:**
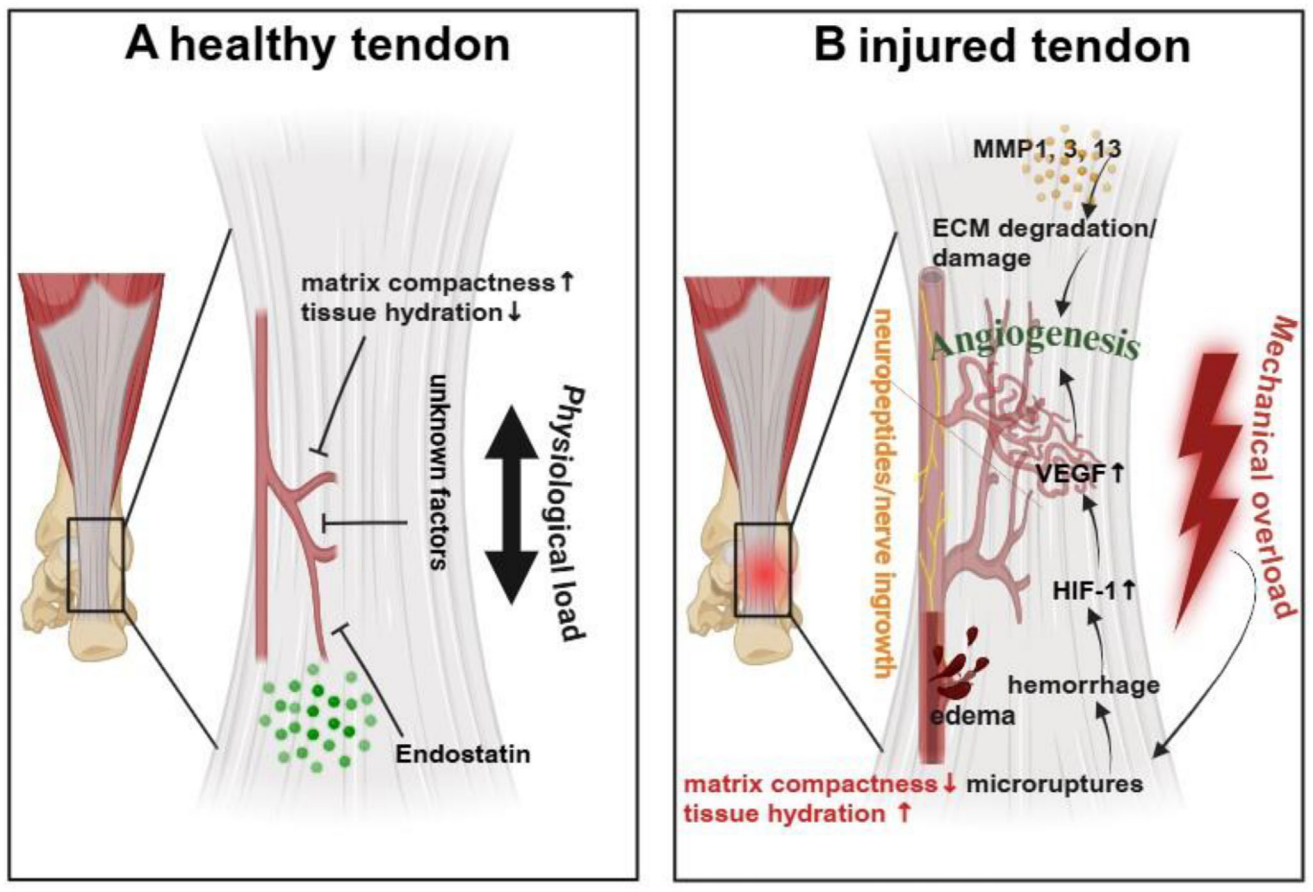
Summary of factors known to maintain the low level of vascularization in healthy tendons. In healthy tendons, limited vascularization is maintained through the expression of anti‐angiogenic factors like endostatin, along with structural features like high matrix density and low tissue hydration. A). In contrast, diseased tendons exhibit increased VEGF expression in response to various stimuli, including hemorrhage, neurogenic mediators, and inflammation. Concurrently, matrix degradation driven by MMPs, along with elevated tissue water content, further promotes blood vessel infiltration B). Created with BioRender.com Adapted with permission.^[^ ^20^ ^]^ Copyright 2025, Elsevier.

The antiangiogenic factor endostatin, a proteolytic fragment of Collagen XVIII, has also been shown to play a role in tendon vascularization. The distribution of endostatin in gliding tendons correlates with the degree of vascularization, and its expression levels are influenced by mechanical load. Specifically, endostatin expression is higher in areas subject to mechanical pressure and reduced in areas without such pressure. This suggests that mechanical input may regulate the expression of antiangiogenic factors.^[^ ^78^ ^]^ Lessons from other sparsely vascularized tissues, such as the cornea, suggest that physical factors like high tissue compactness and dehydration contribute to maintaining an avascular environment,^[^ ^79^ ^]^ besides molecular contributors like soluble VEGF receptors and VEGF inhibiting miRNAs (**Figure** **6**).

Together, these factors may contribute to creating a microenvironment that actively resists vascular ingrowth, preserving the specialized function and structure of the tendon. Elucidating these regulatory pathways in healthy tendon could have important implications for understanding tendinopathies and for designing targeted therapeutic strategies.

## 4 Pathomechanisms

Currently, there is no optimal animal or theoretical model available addressing all features of tendinopathy, such as pain, matrix remodeling, inflammation or angiogenesis. Several theories have been proposed to explain the pathogenesis of tendinopathy, including mechanical, inflammatory, apoptotic, vascular, and neurogenic theories. As their names imply, these theories attribute the primary trigger for tendinopathy to factors such as excessive mechanical loading, inflammatory processes, oxidative stress due to repetitive strain, or increased vascular ingrowth and neurogenic inflammation.^[^ ^1^ ^]^ Additionally, genetic predisposition and the use of certain medications, such as fluoroquinolones, have been identified as potential risk factors.

In 2009 Cook and Purdam introduced the continuum model, which integrates clinical symptoms with structural characteristics observed in histological and imaging analyses to inform treatment decisions.^[^ ^80^ ^]^ This model conceptualizes tendinopathy as a progressive condition, transitioning from a healthy state to degenerative tendinopathy through a reactive stage. At this stage, therapeutic intervention can still facilitate recovery; however, if left untreated, the condition may progress to tendon disrepair and ultimately lead to chronic tendinopathy. The continuum model of tendinopathy offers a useful clinical framework by describing tendon pathology as a progression through reactive, disrepair, and degenerative stages. However, it faces several limitations. The model lacks direct longitudinal evidence and reliable biomarkers to clearly differentiate stages in vivo, making clinical application somewhat subjective. It also oversimplifies the complex, often nonlinear nature of tendinopathy and does not fully account for systemic, neurogenic, or immune factors involved in tendon pain. While valuable, the model should be seen as a conceptual guide rather than a definitive representation of disease progression.

In the following sections we focus on three main drivers considered crucial for the development of tendinopathy: mechanical stress, inflammation and tendon vascularity. By looking into the cellular and molecular mechanisms so far known, we aim to elucidate how these pathways and processes are interconnected and influence one another.

### 4.1 Mechanical Stress

#### 4.1.1 Mechanical Stress‐Induced Pathomechanisms

**Figure 7:**
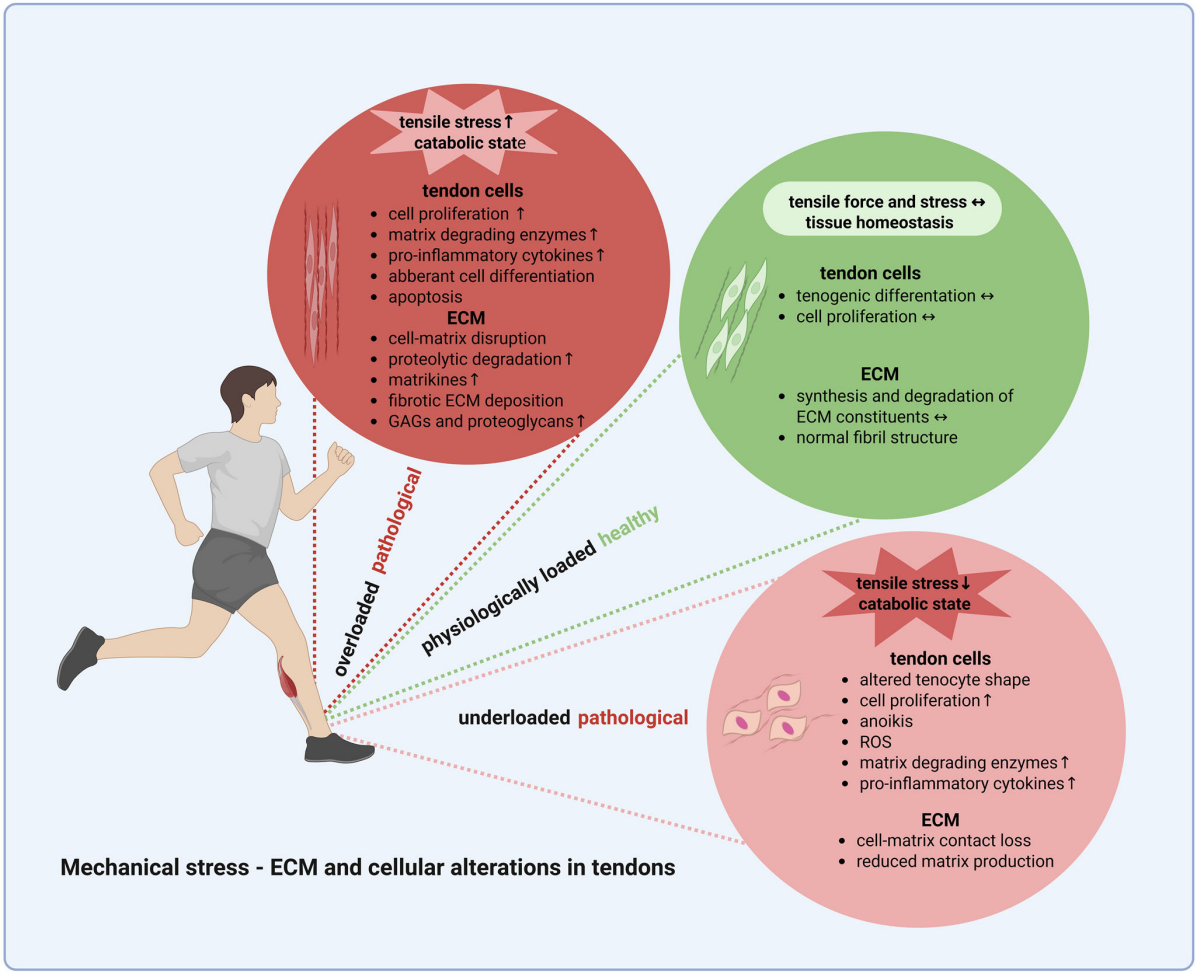
Schematic summary of the key pathological alterations in tendon ECM and cells caused by mechanical overloading and underloading. Created with BioRender.com Adapted with permission.^[^ ^20^ ^]^ Copyright 2025, Elsevier.

Mechanical stress is certainly one of the main drivers of tendinopathy. The mechanical loading of tendons varies depending on factors such as anatomical location, species, age, and gender.^[^ ^81^, ^82^ ^]^ While physiological loads can drive anabolic adaptations in tendons, repetitive loading, overloading, or unloading may lead to catabolic responses, resulting in diseased conditions as shown in **Figure** **7**.^[^ ^83^, ^84^ ^]^

Mechanical load induces a molecular signaling cascade conveying external stimuli to the cell. This cellular mechanosignalling is a dynamic and highly adaptable process that enables cells to constantly sense and interact with their surrounding ECM. This intricate mechanism involves a complex molecular machinery being a critical link between the ECM and the cell itself.^[^ ^85^ ^]^ Mechanotransduction, the process by which cells detect and convert mechanical signals into biochemical responses, is heavily reliant on the cytoskeleton, which acts as a conduit linking external mechanical inputs to intracellular force‐sensing molecules. Cells are capable of rapidly responding to mechanical stimuli such as tension or shear stress by remodeling their actin cytoskeleton. This cytoskeletal remodeling impacts on sensory proteins located within the cell membrane and nucleus, transmitting mechanical signals into biochemical responses that can modulate, e.g., gene expression or protein modification.^[^ ^30^, ^86^ ^]^ As the ability of cells to sense and respond to mechanical cues is essential for their proper functioning, changes in tissue mechanical properties or impairments in cellular mechanosensitivity can be linked to numerous pathological conditions.^[^ ^87^ ^]^ For instance, ECM stiffening observed in fibrosis (scarring) or during aging can compromise cellular responses to mechanical stimuli, leading to alterations in cell motility, proliferation, adhesion, and differentiation.^[^ ^88^ ^]^

Proper mechanical stimulation is indispensable for tendon development, maturation, homeostasis and repair; however, the precise mechanisms by which tendon cells sense ECM‐derived mechanical inputs, the downstream effector molecules involved and how these processes influence physiological and pathological adaptations remain insufficiently understood.^[^ ^88^, ^89^ ^]^ Several potential modes of mechanotransduction in tendon cells are discussed below, some of which are still speculative and require further experimental evidence.

- Tendon cells sense their environment through receptor‐ligand interactions involving soluble factors released by tenocytes or the ECM. These receptors, upon binding to their ligands, transduce mechanical forces via molecular switches such as phosphorylation, activating downstream signaling pathways. Growth factors like TGF‐β, vascular endothelial growth factor (VEGF), fibroblast growth factor (FGF), insulin‐like growth factor (IGF), platelet‐derived growth factor (PDGF), and growth differentiation factor (GDF)‐5/‐6/‐7, as well as cytokines, chemokines, and prostaglandins, are key mediators of mechanotransduction in tendons.^[^ ^90^, ^91^ ^]^ Overuse of tendons often increases the expression of mechanically induced growth factors like TGF‐β, which promotes cell proliferation, migration, and collagen synthesis. However, excessive TGF‐β production may result in collagen overproduction and tendon fibrosis. Similarly, IGF exists as an inactive precursor in healthy tendons but is enzymatically activated in overloaded or injured tendons, stimulating ECM synthesis and cell proliferation via PI3K/AKT and ERK signaling pathways.^[^ ^92^ ^]^
- Mechanosensitive and stress‐activated ion channels are another critical mechanism. These channels respond to changes in membrane tension or tissue deformation, such as stretching, compression, or shear forces, and to osmotic shifts. A prominent group of such channels in musculoskeletal tissues is the transient receptor potential (TRP) channel family, including TRPV (vanilloid), TRPA (ankyrin), and TRPP (polycystic) channels.^[^ ^93^ ^]^ Although their role in tendon mechanotransduction is not fully understood, they likely subserve a similar function as in other musculoskeletal tissues. This might also apply for the heat sensor TRPV1, given temperatures up to 45 °C reached in the tendon core during high gallop in race horses.^[^ ^94^ ^]^
- Recent studies have highlighted the role of PIEZO1, a mechanosensitive channel upregulated in tendon cells under shear stress.^[^ ^95^ ^]^ PIEZO1 activation induces transient calcium influx, promoting collagen cross‐linking enzyme expression, which stiffens the tendon matrix without triggering uncontrolled collagen synthesis. This calcium signaling is strain‐dependent, operating via a feedback loop to reduce cellular responses to mechanical stress.^[^ ^96^, ^97^ ^]^
- Tendon cells also sense mechanical inputs through focal adhesions (FAs), which are dynamic protein complexes linking the actin cytoskeleton to the ECM via integrins.^[^ ^91^, ^98^ ^]^ Adhesion to the ECM or mechanical input induces focal adhesion maturation and conformational changes, driving intracellular signaling pathways such as the Hippo, PI3K/AKT, and Rho GTPase pathways.^[^ ^85^, ^99^ ^]^ Studies have shown that matrix stiffness influences tendon cell responses, with stiffer matrices activating pathways related to chromatin remodeling, Hippo signaling, and cytoskeletal organization, while softer matrices promote ECM modulation and cytoskeletal dynamics. ERK1/2 has emerged as a critical regulator preventing matrix degradation in the absence of mechanical tension, and focal adhesion kinase (FAK)‐ERK signaling modulates cell proliferation and differentiation in response to stiffness changes.^[^ ^99^, ^100^ ^]^ Finally, in a recent study Huang et al. show that cyclic mechanical stretch on tendon cells induces Ca^2+^‐signaling regulated by the AMPK/EGR1 pathway and FAK signaling reinforced cytoskeletal organization due to mechanical input.^[^ ^101^ ^]^

**Figure 8:**
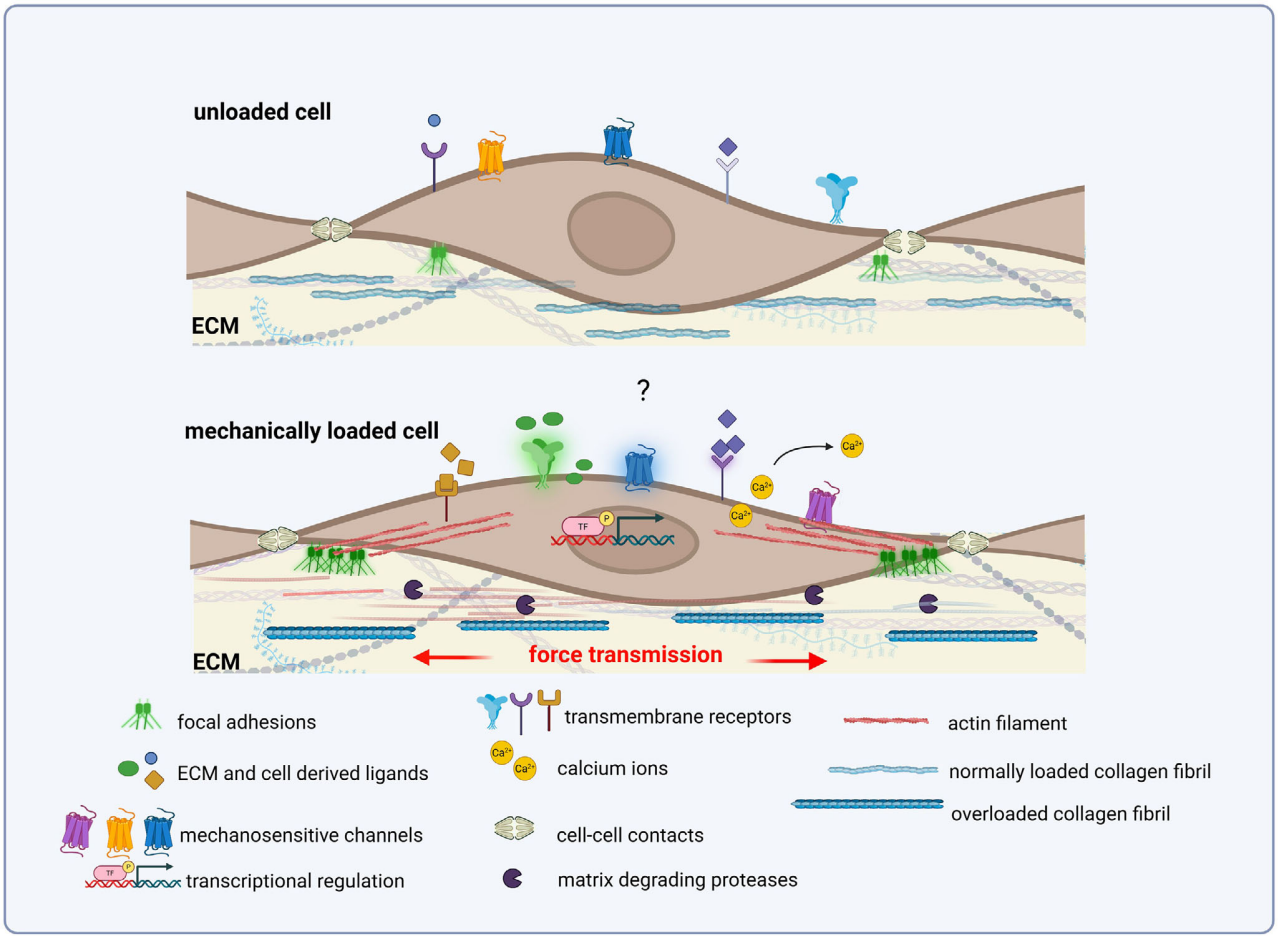
A schematic representation comparing an unloaded cell with a mechanically loaded cell, highlighting the various molecular components involved. Next to receptor–ligand interactions, mechanosensitive and stress‐activated ion channels, focal adhesion complexes are key mediators of transducing force displacement into cellular signals like transcriptional changes, alterations in calcium concentration, or release of matrix degrading proteases. Created with BioRender.com Adapted with permission.^[^ ^20^ ^]^ Copyright 2025, Elsevier.

Alternatively, cells can sense their environment through other transmembrane receptor proteins that directly bind specific ECM components. For instance, collagen is recognized by discoidin domain receptors,^[^ ^102^ ^]^ while CD44 facilitates cell adhesion in hyaluronan‐rich matrices.^[^ ^103^ ^]^ **Figure** **8** summarizes the diverse mechanisms by which tendon cells sense mechanical force and interact with the surrounding extracellular matrix.

The biomechanical properties of the tendon ECM significantly influence how tendon‐resident cells respond to mechanical stimuli. Among the emerging factors, SPARC has gained attention for its role in tendon mechanobiology. SPARC is essential for load‐induced tendon maturation, with SPARC‐null mice showing catabolic tendon phenotypes under loading conditions and increased susceptibility to spontaneous tendon tears.^[^ ^104^ ^]^ Functional calcium imaging has demonstrated that lower stress thresholds are required to activate mechanically induced responses in SPARC‐deficient tendons. Its pleotropic functions further include interaction with integrins, activating intracellular signaling pathways involving AKT, FAK, and integrin‐linked kinase (ILK).^[^ ^105^, ^106^ ^]^

Conversely, cells actively influence their ECM through three primary mechanisms. First, they modify ECM composition by depositing new matrix components, secondly, they secrete matrix‐remodeling enzymes adjusting the matrix structure and thirdly, cells apply traction forces stiffening the pericellular matrix. This dynamic interplay between cells and the ECM is critical for tissue homeostasis and functionality, alterations in any of these processes can lead to pathological changes. Given that tendons are load‐bearing tissues, tendon cell function is highly dependent on continuous mechanical stimulation.^[^ ^107^ ^]^ As mentioned, tendon cells adapt their extracellular environment by modifying ECM composition or secreting matrix‐degrading enzymes after sensing varying mechanical inputs. Furthermore, tendons cannot be considered in isolation; they are a part of the muscle‐tendon unit, connecting muscle to bone through specialized interfaces. This connection creates regional variations in composition and mechanical properties between transition zones and intratendinous regions, adding complexity to tendon mechanobiology and making it a challenging area to study.^[^ ^89^ ^]^

Overall, tendon pathology develops when mechanosignaling shifts from an adaptive to a maladaptive response. Rather than supporting healing, it drives chronic remodeling, inflammation, and fibrosis through key players like integrin–FAK, TGF‐β/SMAD, YAP/TAZ, and ion channels. The result is a tendon that becomes mechanically dysfunctional, fibrotic, and painful.^[^ ^89^, ^108^ ^]^

In summary, mechanosignaling in tendons involves multiple interlinked pathways that do not function in isolation. There is increasing evidence that these pathways converge on common cellular processes, forming an integrated mechanoresponsive network. While they originate from different sensors (e.g., integrins versus stretch‐activated ion channels), they converge at the level of shared transcriptional targets, cytoskeletal regulation, and matrix remodeling, allowing the tendon to mount a coordinated but adaptable response to mechanical stress. However, spatial, temporal, and mechanical context shapes their activity and downstream effects, allowing for both convergence and distinction.^[^ ^109^, ^110^, ^111^ ^]^

#### 4.1.2 Mechanical Load‐Induced Alterations of Vasculature

The integrity of vascular barriers is regulated by numerous well‐characterized factors, some of which are mechanosensitive. One such factor is VEGF, originally identified as “vascular permeability factor” due to its ability to induce endothelial hyperpermeability, cell proliferation, angiogenesis, and enhanced glucose transport.^[^ ^112^ ^]^ Mechanical stimulation has been shown to upregulate VEGF expression in tendon cells, for instance, applying cyclic mechanical strain (1 Hz, 10% strain) in vitro significantly increased VEGF levels, indicating that mechanical loading can directly drive VEGF production in. tendon tissue^[^ ^113^ ^]^

Bradykinin is another mediator implicated in vascular permeability. This peptide, known for its role in inflammation, vasodilation, and pain sensitization, enhances vascular permeability, and promotes the release of other inflammatory mediators. Langberg et al. demonstrated that mechanical loading during exercise elevated bradykinin levels in the peritendinous space of the Achilles tendon and muscle, suggesting a load‐dependent increase in bradykinin that may influence local vascular permeability.^[^ ^114^ ^]^

**Figure 9:**
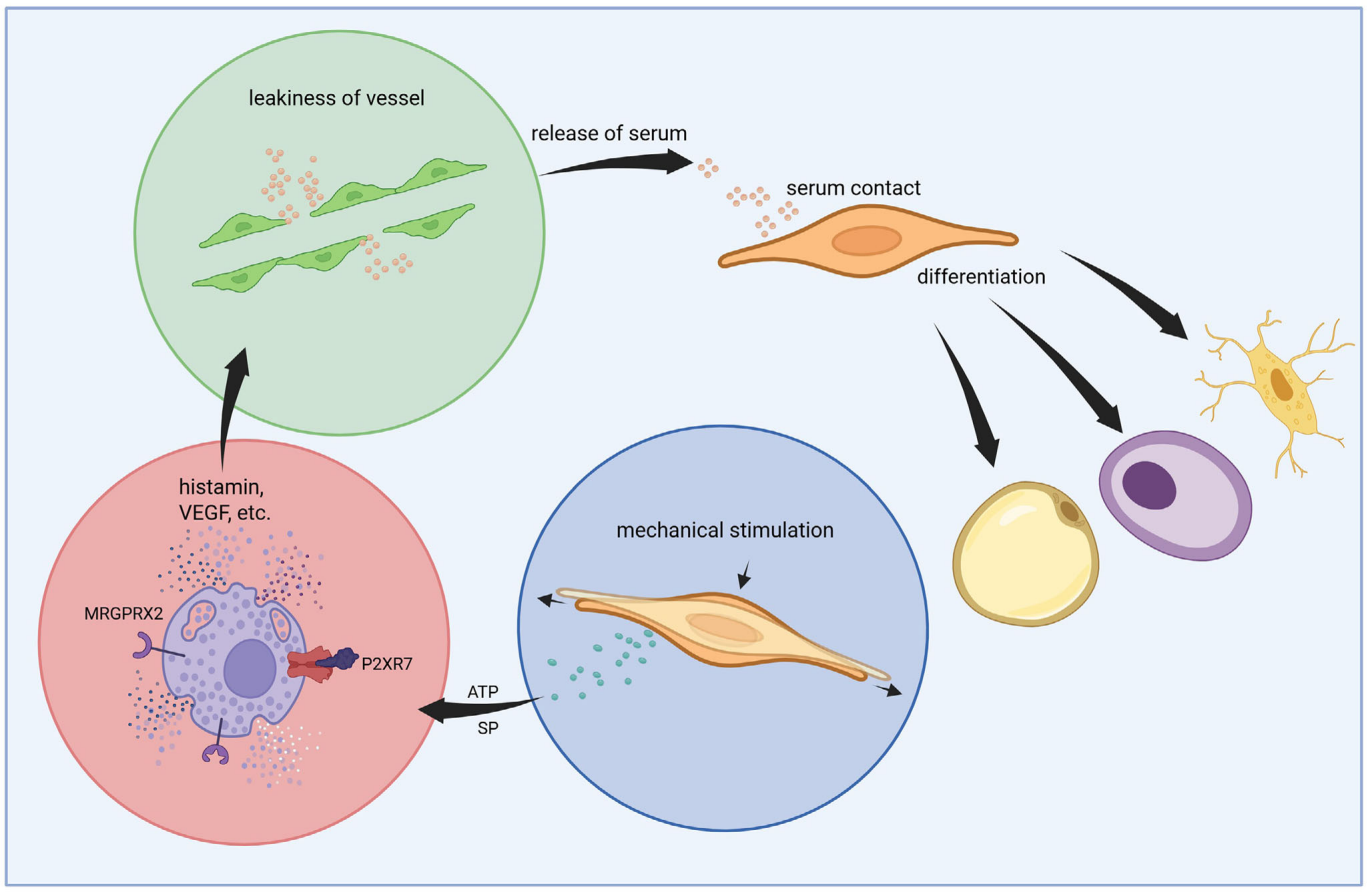
Proposed mechanism of mast cell‐mediated tendon cell differentiation: Mechanically stimulated tendon cells release ATP and Substance P, which bind to specific receptors on mast cells, triggering their degranulation. The resulting release of mediators enhances blood vessel permeability and promotes serum extravasation, creating a microenvironment that facilitates the differentiation of tendon cells into chondrogenic, adipogenic, and osteogenic lineages. Created with BioRender.com.

Histamine also plays a central role in increasing vascular permeability by acting on endothelial H1 receptors. This interaction activates RhoA/ROCK signaling, leading to cytoskeletal changes and the opening of intercellular junctions, thereby allowing plasma proteins and fluids to extravasate into tissues and contribute to inflammation.^[^ ^115^ ^]^ Mast cells, the primary source of histamine, are present in tendon tissue and are more abundant in tendinopathic conditions.^[^ ^116^, ^117^ ^]^ Mechanical stress can stimulate tenocytes to release substance P, which in turn activates mast cells via the MRGPRX2 receptor, triggering histamine release and potentially exacerbating local inflammation and vascular permeability.^[^ ^118^ ^]^ Moreover, mechanically stressed tendon cells release ATP which in turn can activate P2XR7 receptors present on mast cells leading to their degranulation and release of histamine and other factors described to compromise blood vessel tightness^[^ ^119^, ^120^, ^121^, ^122^ ^]^ (**Figure** **9**).

However, a direct mechanistic effect of tendon overuse on reduction of blood vessel tightness and thus unfavorable vessel leakage has not been shown yet, representing a clear research gap.

#### 4.1.3 Mechanical Load‐InducedInflammation

In tendons, mechanical stress and inflammatory processes are closely interconnected in various aspects. First, mechanical stress on collagen creates mechanoradicals by homolytic bond scission and subsequently leads to the generation of reactive oxygen species (ROS) and oxidative stress which are closely linked to inflammation response which is comprehensively detailed in a recent review by Yu et al.^[^ ^123^, ^124^, ^125^ ^]^

Second, inflammatory signaling has been shown to sensitize Piezo1‐mediated mechanotransduction in articular chondrocytes, contributing to a pathogenic feed‐forward loop in osteoarthritis. Similarly, inflammatory mediators enhance Piezo2‐dependent mechanosensitive currents in sensory neurons—a mechanism that is likely relevant in tendon cells as well.^[^ ^97^, ^126^ ^]^

Third, tenocyte cell death induced by mechanical stress not only leads to the release of cytokines including IL1β, but also to Toll‐like receptor (TLR)‐activating danger‐associated molecular patterns (DAMPs) which in turn can induce the expression of inflammatory molecules such as IL1β, IL6 and chemokine CC‐Chemokin‐Ligand‐2 (CCL2).^[^ ^127^, ^128^ ^]^ Interestingly, not only mechanical overload, but also underloading of tendon cells leads to a release of inflammatory cytokines including IL1β, TNFα, and TGFβ.^[^ ^129^, ^130^ ^]^ For an in‐depth discussion on how pathological mechanical loading acts as a biomechanical stressor that triggers immune‐mediated tissue repair pathways in tendons and ligaments, readers are referred to the comprehensive review by Gracey et al., which also explores shared immunopathogenic mechanisms between tendinopathy and select forms of chronic inflammatory arthritis.^[^ ^131^ ^]^

Collectively, understanding the mechanobiological circuits governing tendon cell responses to physiological and pathological loading is advancing, but remains an area of intensive research.

### 4.2 Inflammation

Although controversially discussed for a long time, it has now been well accepted that inflammatory processes contribute to the development of tendinopathy. Meanwhile, the proven presence of immune cells in healthy and diseased tendons as well as reports on a plethora of cytokines being involved in disease onset and progression, underpin this concept.^[^ ^116^, ^132^, ^133^, ^134^, ^135^, ^136^ ^]^

#### 4.2.1 Local and Systemic Inflammation

In tendons and ligaments, tenocytes play a key role in sensing mechanical stress through specialized receptors and mechanisms mentioned earlier on. They respond by modulating the extracellular matrix (ECM) to maintain tissue homeostasis. Both insufficient mechanical stimulation and excessive loading can trigger proinflammatory responses, contributing to the development of tendinopathy.

Consistently, ex vivo mechanical loading of mouse Achilles tendons and tenocytes within the physiological range showed no significant differences in IL‐1β levels between loaded and unloaded controls, and even a notable downregulation of TNFα.^[^ ^137^ ^]^ However, when mechanical stress exceeds physiological thresholds—either due to overload or deprivation—tendon‐resident cells release pro‐inflammatory cytokines such as IL‐1β and TNFα.^[^ ^129^, ^138^ ^]^ Interestingly, the addition of TNFα to human tenocytes in vitro led to a significant upregulation of MMP‐1, along with increased expression of pro‐inflammatory (TNFα, IL‐1β) and immunoregulatory (IL‐6, IL‐10) cytokines.^[^ ^139^ ^]^ This suggests that inflammatory cytokines released by stressed tendon or immune cells drive tenocytes to further amplify pro‐inflammatory signaling, creating a degenerative feedback loop. This degenerative process is further exacerbated by the upregulation of proteases under inflammatory conditions. Various matrix metalloproteinases (MMPs), along with members of the cathepsin and ADAMTS families, have been shown to be elevated in inflammatory environments, where they serve as key mediators of collagen degradation and ECM breakdown.^[^ ^140^, ^141^ ^]^ Notably, these catabolic enzymes are not produced solely by tendon cells, immune cells being a primary source of cathepsins. A recent review has detailed the role of various cathepsin family members in inflammatory disorders across multiple organs, including the brain.^[^ ^142^ ^]^

Next to local tissue inflammation, elevated systemic cytokine levels have been shown to impact on tendon quality. In a comprehensive review, Abate et al. (2013) studied the occurrence of tendon pathologies in metabolic disorders including adiposity, diabetes, hypercholesterolemia, and chronic gouty arthritis.^[^ ^143^ ^]^ That tendon disease very likely is related to the subclinical persistent inflammation going along with these metabolic disorders has been demonstrated by a recent study.^[^ ^144^ ^]^ By using an allergy mouse model it has been shown that elevated blood cytokine levels lead to an increase in tendons′ cross sectional area and impairment of their biomechanical properties, significantly reducing the Young's modulus and tensile stress.^[^ ^144^ ^]^ Interestingly, the mere presence of a systemic inflammation for a relatively short time period, can impact tendon ECM integrity as evidenced by increased levels of MMP2 expression and an increased expression of immune cell related markers such as F4/80, CD68, and CD163.

**Figure 10:**
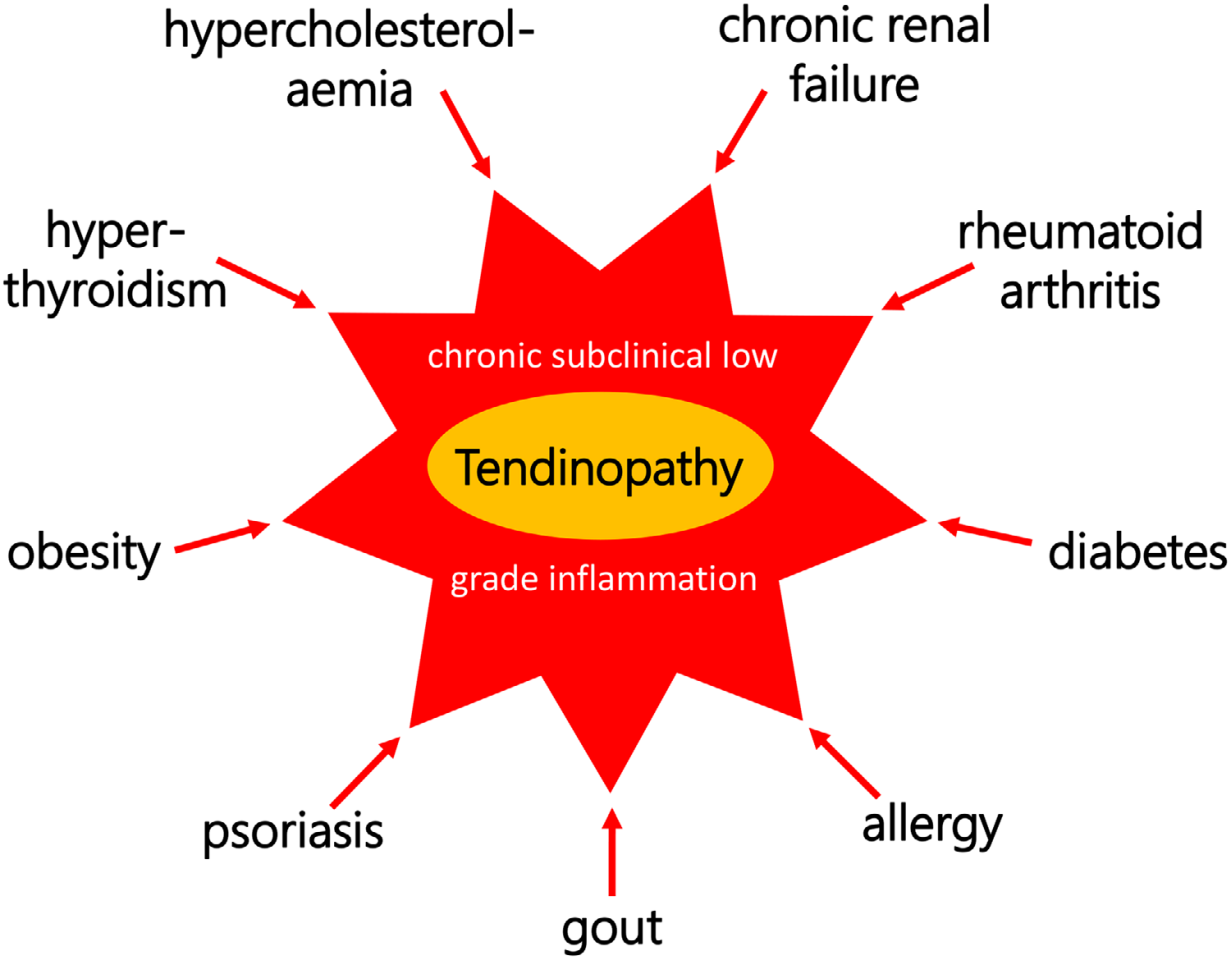
Diseases known to be associated with chronic systemic inflammation and tendinopathy.

Diseases so far described to be associated with the occurrence of spontaneous ruptures or tendinopathic symptoms are summarized in **Figure** **10**. How the presence of a systemic subclinical inflammation and biomechanical signals might act together has been convincingly shown by Cambré et al (2018). In their study, the authors addressed the reason behind the regional and patchy distribution of arthritis that is known to be a systemic inflammatory disease and provide the first evidence that biomechanical loading is a decisive factor in the transition from systemic to site‐specific inflammation, a mechanism that could also apply for tendinopathy.^[^ ^145^ ^]^

#### 4.2.2 Interplay between Immune Cells and Tenocytes

The onset of tendinopathy is still far from understood, but it seems that cross talk between different tenocyte subpopulations, as well as between these subtypes and different immune cell types present in the tendon, plays a crucial role.

Thereby, one has to keep in mind that number and localization of immune cells differs between healthy and tendinopathic tendons. Whereas in intact tendons macrophages and mast cells are located in close proximity to blood vessels, and are almost not present in the dense part of the tissue, the situation changes under diseased conditions. Alongside the sprouting and ingrowth of blood vessels, blood‐borne immune cells migrate from the circulation to the site of injury, thus, intensifying the interaction between immune cells and tendon cells. In this context, the aforementioned CX3CR1/CX3CL1 receptor‐ligand axis might play a role in attracting monocytes/macrophages from the blood stream to the tissue.

Several in vitro studies have demonstrated the crosstalk between tenocytes and immune cells. By using conditioned media from IL‐1ß‐ activated tenocytes and performing coculture experiments Garcia‐Melchior et al. (2021) showed induced migration and activation of T cells which, in turn, led to increased production of inflammatory mediators in tenocytes.^[^ ^27^ ^]^

Similarly, in another study it has been shown that tenocytes respond to inflammatory environments (INFγ, TNFα, IL1β) not only with altered surface marker and cytokine profiles, but also a change in macrophage polarization.^[^ ^146^ ^]^ Additionally, co‐cultures between macrophages and prestimulated tenocytes were shown to release increased levels of IL‐6, IL‐8, MCP‐1 than tenocyte‐cultures alone. Interestingly, similar results have been found in a transwell setting implicating paracrine cell communication.

Stauber et al. also demonstrated crosstalk between immune cells and tenocytes using an in vitro tendon model composed of mouse tail tendon fascicles encased in cell‐laden collagen hydrogels, which mimic the tendon core and the surrounding extrinsic tissue compartment, respectively.^[^ ^147^ ^]^

Interestingly, a study by Akbar et al. indicated a dysregulated immune homeostasis in diseased tendons.^[^ ^148^ ^]^ By performing cell‐cell interaction analysis the authors established an atlas of the dynamic cellular environment that drives the development of chronic human tendon disease. Pathway analysis of stromal cells derived from healthy and diseased human tendons revealed an apparent shift from negative regulation of immune cells and cytokine responses in intact tendons to an immune cell activating state in diseased tendon, likely promoting a cycle of inflammation and aberrant tissue repair.

#### 4.2.3 Acute versus Chronic Inflammation versus Resolution

The primary objective of the early inflammatory phase is to clear waste and debris following injury, facilitating the restoration of normal tissue structure and function. This process can occur through either resolution, which restores tissue morphology to its original state, or repair, which leads to functional recovery but with structural alterations.

**Figure 11:**
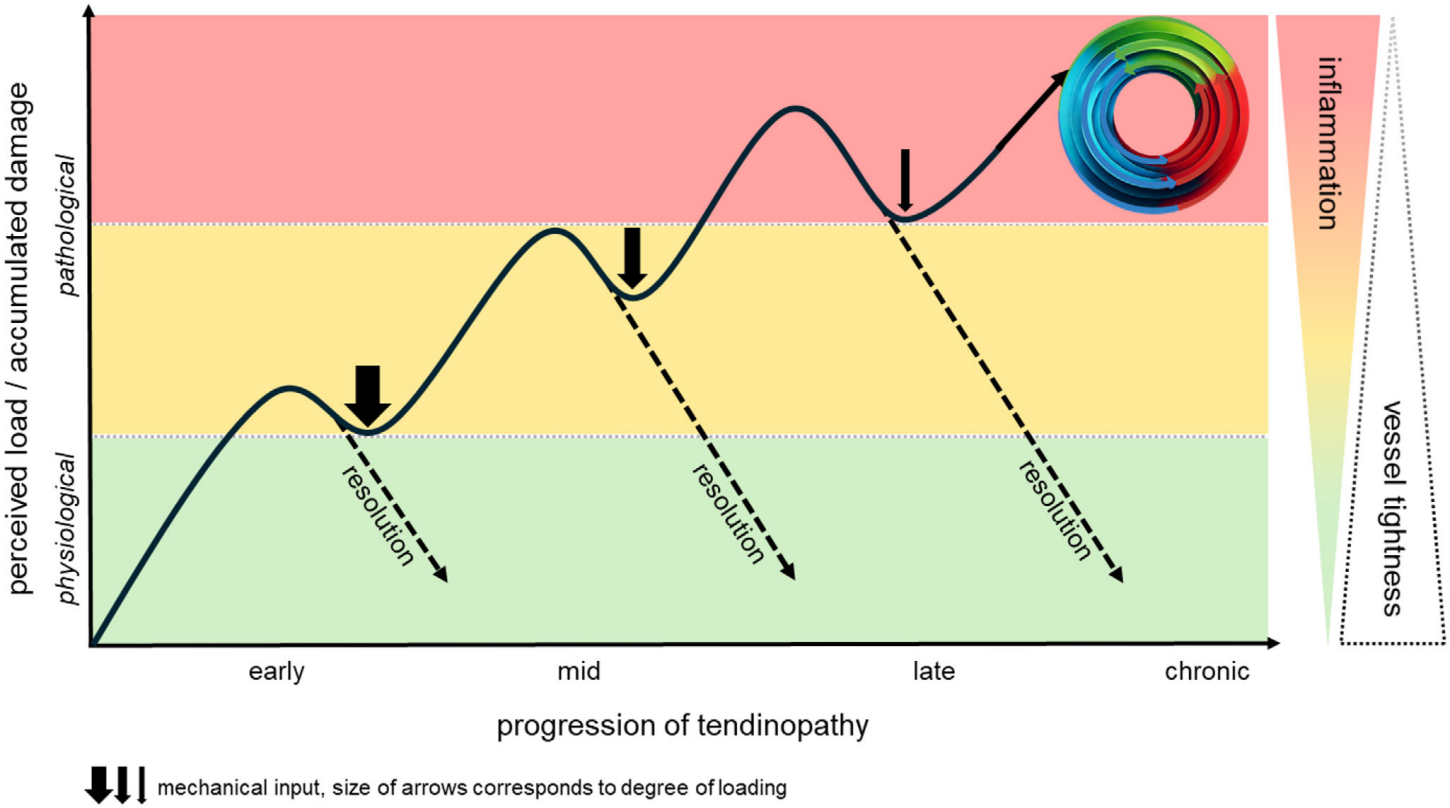
Schematic illustrating tendinopathy progression: When an overloaded tendon does not have sufficient time to repair accumulated damage before encountering a new mechanical stimulus, the resulting inflammatory environment sensitizes mechanoreceptors such as PIEZO1, lowering the threshold for mechanical input needed to elicit the same cellular response. If this occurs repeatedly over time, the condition progresses to a chronic state, entering the tendinopathic cycle. Created with BioRender.com.

In acute inflammation, tissue damage is typically resolved; however, in chronic inflammation, ongoing tissue damage counteracts the repair process. While the initial inflammatory response is generally acute, it can transition into chronic inflammation if the underlying inflammatory triggers persist and resolution mechanisms fail (**Figure** **11**).

Among the key pro‐resolving mediators identified in this process are 15‐epi‐Lipoxin A4 (15‐epi‐LXA4) and maresin (MaR1), both of which have been shown to play active roles in Achilles tendinopathy and tendon repair.^[^ ^149^ ^]^ These mediators not only enhance the expression of specialized proresolving mediator (SPM) biosynthetic enzymes, ALOX12 and ALOX15, as well as the pro‐resolving receptor ALX, but also modulate the inflammatory phenotype of Achilles tendon stromal cells by suppressing pro‐inflammatory signaling pathways. Consistently, the expression of the FPR2/ALX receptor on tenocytes was significantly elevated in sub‐acute but not chronically injured equine superficial digital flexor tendons (SDFT). Meanwhile, its ligand, Annexin A1, showed a marked increase in both injury conditions, in contrast to its low expression levels in healthy tendons.^[^ ^150^ ^]^

Taken together, if healing mechanisms are dysregulated and inflammation exacerbates, then tissue damage progresses, ultimately resulting in chronic tendinopathy.

#### 4.2.4 Hypoxia and Inflammation

Due to the inherently low vascularity of tendons, high mechanical stresses that require increased metabolic activity and ATP consumption are likely to create hypoxic conditions. Additionally, microtears resulting from overload trigger an inflammatory response, involving pro‐inflammatory cytokines such as IL‐1β and TNF‐α. These cytokines have been shown to directly influence HIF signaling by regulating HIF expression at both transcriptional and post‐translational levels.^[^ ^151^ ^]^ In turn, hypoxia, through the activation of HIF‐1α, has been shown to directly regulate the production of pro‐inflammatory cytokines such as TNF‐α, IL‐1β, IL‐6, and IL‐8 in synovial fibroblasts of rheumatoid arthritis patients.^[^ ^152^ ^]^ This interplay between inflammatory and hypoxic signaling pathways is essential in regulating both the nature and intensity of inflammation at hypoxic sites. Accordingly, hypoxia is recognized as a key factor in the early stages of human tendinopathy, as it stimulates the production of pro‐inflammatory cytokines, key apoptotic mediators, and shifts matrix synthesis in human tenocytes toward a collagen type III profile.^[^ ^77^, ^153^ ^]^

#### 4.2.5 Metabolism and Inflammation

Traditionally, metabolism and inflammation have long been viewed as two separate domains within biology. Recent research has revealed that these two processes are intricately intermingled and impact each other which led to the coining of the new term “immunometabolism”.^[^ ^154^ ^]^ On one side, ongoing inflammatory and immune responses are associated with dramatic shifts in tissue metabolism due to an increased consumption of nutrients, and oxygen, as well as generation of ROS and oxygen intermediates by proliferating immune cells. On the other hand, dietary and lifestyle factors have ramifications on cellular metabolism, which in turn modulates the nature of the inflammatory response toward either a proinflammatory state or the resolution of inflammation.^[^ ^155^ ^]^ Furthermore, metabolic danger‐associated molecular patterns (DAMPs) such as LDL cholesterol, fatty acids, and hyperglycemia have implications on inflammation since they “train” immune cells which up‐or downregulates innate immune cell activity.^[^ ^156^ ^]^

### 4.3 Tendon Vascularity

One of the main clinical features of chronic tendinopathy is increased angiogenesis, which may even serve as a diagnostic hallmark.^[^ ^157^ ^]^ However, the underlying mechanisms are still poorly understood.

#### 4.3.1 Mechanisms Leading to Hypervascularization in Tendon Pathology

Following tendon injury, neoangiogenesis occurs as a physiological response that supplies the injured site with oxygen and facilitates the infiltration of inflammatory cells. This process also supports the resorption of damaged material during later stages of inflammation and tissue repair. VEGF plays a central role in driving neoangiogenesis in tendon diseases and injuries. However, the mechanisms leading to VEGF upregulation and angiogenesis are multifaceted and may involve mechanical stimulation, inflammation, neurogenic signals, and hypoxia, but can also be triggered by neurogenic stimulation.^[^ ^158^ ^]^

In vitro studies show that mechanical overload, applied at 8% strain to cultured rat tendon cells, increases the expression of VEGF isoforms in a frequency‐dependent manner. Additionally, levels of HIF1α are also elevated following cyclic loading.^[^ ^159^ ^]^ Similarly, applying 10% strain cyclic loading to human tendon cells results in increased expression of multiple angiogenic genes, including ANGPTL4, FGF‐2, COX‐2, SPHK1, TGF‐α, VEGF‐A, and VEGF‐C. Notably, this process does not alter mRNA levels of antiangiogenic factors such as BAI1, SERPINF1, THBS1, and TIMP1‐3.^[^ ^113^ ^]^ Interestingly, the mechanosensitive ion channel Piezo1 has been shown to regulate sprouting angiogenesis through the activation of membrane‐type 1 matrix metalloproteinase (MT1‐MMP) signaling. Genetic deletion or pharmacological inhibition of Piezo1 reduces endothelial sprouting, whereas activating Piezo1 with the selective activator Yoda1 enhances sprouting angiogenesis.^[^ ^160^ ^]^

The role of inflammatory signals in angiogenesis is well‐studied across various tissues. In tendons, inflammatory cytokines are highly expressed following acute injury and during tendinopathy.^[^ ^161^ ^]^ Along similar lines, CX3CL1 (Fractalkine), a chemokine shown to act as an angiogenic mediator, is upregulated in tendons under pathological conditions. It exerts proangiogenic effects in vitro on human umbilical vein endothelial cells and promotes corneal neovascularization in vivo.^[^ ^162^ ^]^

Hypoxia, which is a lack of oxygen supply to tissues, is a major factor inducing new blood vessel formation. Samples from human rotator cuff tendons in the early stages of tendinopathy exhibit signs of hypoxia, including upregulation of HIF1α. Tendon cells cultured under hypoxic conditions respond by increasing VEGF expression and undergoing apoptosis.^[^ ^77^ ^]^ Despite this, the actual oxygen availability in healthy tendons and how it changes with disease remain poorly understood.

The observation that explanted tendons swell when immersed in physiological buffer solutions indicates that tendons may be underhydrated in vivo.^[^ ^163^ ^]^ Inflammation and mechanical damage can lead to edema, disrupting this balance and creating a less antiangiogenic environment. Furthermore, matrix degradation due to upregulated MMPs in tendon inflammation reduces tissue compactness, potentially facilitating not only the ingrowth of blood vessel but also of nerves.^[^ ^70^ ^]^

Pain is a primary clinical symptom of tendinopathy and may result from nerve ingrowth which is often associated with overuse damage and inflammation. Neuropeptides such as Substance P and CGRP are upregulated in damaged tendon tissues and are known to enhance pain.^[^ ^164^ ^]^ These neuropeptides, along with others like noradrenaline, glutamate, and neuropeptide Y, not only affect pain perception and neuron sensitization, but also influence angiogenesis. Substance P directly promotes tendon cell proliferation and angiogenesis when injected, while CGRP is similarly implicated in blood vessel formation. Indirectly, Substance P and CGRP stimulate mast cells to release histamine, which subsequently causes vasodilation and angiogenesis.^[^ ^7^, ^165^ ^]^

#### 4.3.2 Blood Vessel Permeability – A Player in Tendon Disease?

Blood vessels regulate tissue supply by controlling oxygen, nutrient, and solute delivery. This regulation depends not only on vascular density and blood flow but also on the permeability of the vessel wall. In endothelial cells, tight junction proteins like ZO1, occludin, claudin 3, and claudin 5 create a barrier that limits the diffusion of molecules across the vessel wall. This “blood‐tendon barrier” has been identified in human and murine tendon endothelial cells, restricting the passage of molecules larger than 10 kDa. Electron microscopy studies show that blood vessels in healthy human tendons are non‐fenestrated.^[^ ^56^, ^166^ ^]^ Despite these insights, it remains unclear how this barrier is altered in tendon disease. Interestingly, in vitro experiments suggest that serum presence affects hallmark characteristics of tendinopathy. First, it has been shown that tendon cell proliferation, a common feature of tendinopathy leading to hypercellularity, decreases in a serum‐dependent manner. Secondly, the ability of tendon cells to undergo osteogenic and adipogenic differentiation appears to be influenced by serum availability, with serum withdrawal leading to a loss of differentiation potential. And thirdly, the expression of collagen‐degrading enzymes MMP2 and MMP9 increases in the presence of serum. These findings imply that alterations in the blood‐tendon barrier could contribute to tendinopathy. However, functional evidence of barrier disruption in diseased tendons is still lacking.^[^ ^56^ ^]^

VEGF, known to promote vascular growth, also increases vessel permeability. Its upregulation in tendinopathy likely compromises blood vessel tightness, particularly in immature, newly formed vessels.^[^ ^167^ ^]^ Inflammation, a key factor in tendinopathy, can also disrupt endothelial barriers, as seen in other diseases like multiple sclerosis and ischemic stroke. Proinflammatory cytokines such as TNF‐α, IL1‐β, and IL6—key players in tendinopathy—have been shown to impair barrier function. Furthermore, leukocyte infiltration into the vessel wall negatively affects vascular integrity.^[^ ^166^ ^]^ These mechanisms may also be relevant to diseased tendons.

In other tissues, such as the brain, pathologic blood vessel leakage similarly leads to tissue damage and impaired function. It can cause serious damage by allowing blood components such as immune cells, cytokines, and plasma proteins like fibrinogen to enter brain tissue, leading to inflammation, neuronal injury, and increased intracranial pressure.^[^ ^168^ ^]^ These factors can alter the local microenvironment, skewing neural stem cell differentiation toward gliogenesis (glial cell production) rather than neurogenesis (neuron formation). Leakage of proteins like fibrinogen activates the TGF‐β pathway in neural stem cells. This has been shown to inhibit neuronal differentiation and promote astrocytic fate, leading to glial scar formation and impaired brain repair. The TGF‐β pathway is a crucial driver of fibrosis in many tissues, thus this mechanism may be also be relevant in tendon disease.

#### 4.3.3 Neovascularization During Tendon Healing – Friend or Foe?

Various well established treatment options for tendinopathy indirectly point toward an involvement of (neo)vessels in the pathophysiology of tendinopathy, even though this indirect evidence is partly conflicting.

A large body of literature supports the positive effects of extracorporeal shock wave (ESWT) on functional and pain‐related outcomes in tendinopathy.^[^ ^169^ ^]^ ESWT has been shown to enhance tendon angiogenesis by inducing VEGF expression through HIF1α stabilization and mobilizing endothelial progenitor cells from the bloodstream. Additionally, ESWT promotes angiogenic responses by stimulating endothelial cell caveolae, and it increases MMP9 expression, which facilitates matrix remodeling allowing vessel ingrowth as well as activation of VEGF.^[^ ^170^, ^171^ ^]^

On the other hand, eccentric loading, a method involving controlled elongation of muscles and tendons, is another effective treatment for tendinopathy. This approach reduces neovessel density in affected areas, which may explain its therapeutic effectiveness. Compression and cryotherapy similarly reduce tendon blood flow and improve outcomes. Low‐level laser irradiation and topical nitroglycerin are also discussed for their effects on tendon microcirculation. While nitroglycerin improves clearance of metabolic products through vasodilation, low‐level laser irradiation may promote microthrombosis or partial destruction of neovessels. However, the mechanisms underlying these therapies remain unclear.^[^ ^70^ ^]^

Polidocanol injections, which have a sclerosing and local anesthetic effect, have demonstrated significant success in treating Achilles tendinopathy by reducing vascularization and alleviating symptoms in nearly all patients in a randomized controlled trial.^[^ ^172^ ^]^ Despite this, some studies report no correlation between the degree of tendon vascularization and pain symptoms.^[^ ^164^ ^]^

These contradictions highlight that the extent of vascular inhibition and the timing of intervention are critical determinants of therapeutic outcomes. Moreover, many treatments targeting tendon vascularization likely affect additional biological processes. For instance, EWST therapy not only induces angiogenesis but also modulates inflammation through mechanisms involving Substance P (SP), CGRP, nitric oxide signaling, and Toll‐like receptor 3 signaling.^[^ ^170^ ^]^ Additionally, shockwave treatment triggers the release of cellular ATP, activating purinergic receptors and promoting tendon cell proliferation via downstream Erk1/2 signaling pathways in vitro and in vivo.^[^ ^173^ ^]^

The complexity of VEGF and its antagonists extends beyond angiogenesis. Both human and rodent tendon cells express VEGF receptors and respond to injury or inflammatory stimulation. Inhibition of VEGF receptor 3 specifically reduced tendon degeneration in an ex vivo rat tail tendon model of underload‐induced damage, underscoring the multifaceted roles of VEGF signaling in tendon biology.^[^ ^72^ ^]^

#### 4.3.4 Lymphatic Drainage of Tendons

Lymphatic drainage in intact tendons has received little attention in the scientific literature. Early reports using India ink injections identified lymphatic vessels associated with blood vessels in calf tendons.^[^ ^14^ ^]^ However, definitive identification of lymphatic vessels generally requires detecting specific markers such as LYVE1, PROX1, Podoplanin, or VEGFR3.^[^ ^174^ ^]^

In healthy tissues, lymphatic vessels serve to drain excess interstitial fluid and soluble molecules. Following injury or during inflammation, these vessels proliferate and play critical roles in immune surveillance. In our studies of intact rat Achilles tendons, we found no evidence of lymphatic vessels. However, lymphatic vessels began to grow into repair tissue following tendon injury, suggesting a role in tendon healing.^[^ ^174^ ^]^

The lymphatic drainage of tendons following injury and in cases of tendinopathy is a promising, yet underexplored area of research that warrants greater attention in both basic and clinical studies, particularly as intratendinous pressure gains recognition as a potential contributor to tendon pathologies and associated pain.^[^ ^175^, ^179^ ^]^ Given the surprisingly limited knowledge about lymphatic drainage in tendons under both healthy and pathological conditions—and the near absence of high‐quality data from relevant animal defect models—we present here an overview of insights from other tissues that share structural or functional similarities with tendon:

In fibrous tissues like the skin, lymphangiogenesis is triggered by inflammation following injury and can be effectively suppressed using anti‐inflammatory agents such as dexamethasone. Although lymphatic vessels are present in healthy skin, they typically remain inactive unless stimulated by pathological conditions.^[^ ^176^ ^]^ Administering lymphangiogenic factors to the skin has been shown to alleviate acute inflammation by enhancing lymphatic flow and reducing edema in a mouse model.^[^ ^177^ ^]^ Similarly, in chronic venous leg ulcers in humans, impaired lymphatic drainage has been observed, which may contribute to delayed wound healing.^[^ ^178^ ^]^ This delay is likely due to elevated tissue pressure stemming from inadequate fluid clearance.

Tendinopathic tissue often exhibits an accumulation of glycosaminoglycans (GAGs), which have strong water‐binding capabilities. This buildup leads to tissue swelling and elevated pressure within the tendon, placing excessive compressive load on resident cells. Such mechanical strain may provoke maladaptive cellular responses that drive disease progression. While still speculative, improving lymphatic drainage could help mitigate both mechanical stress and fluid retention in tendinopathy. By doing so, it may be possible to reduce excessive compression, potentially slowing disease progression and relieving pain.^[^ ^180^ ^]^ The role of lymphatic drainage in tendon healing and tendinopathy remains a promising yet underexplored research area.

**Figure 12:**
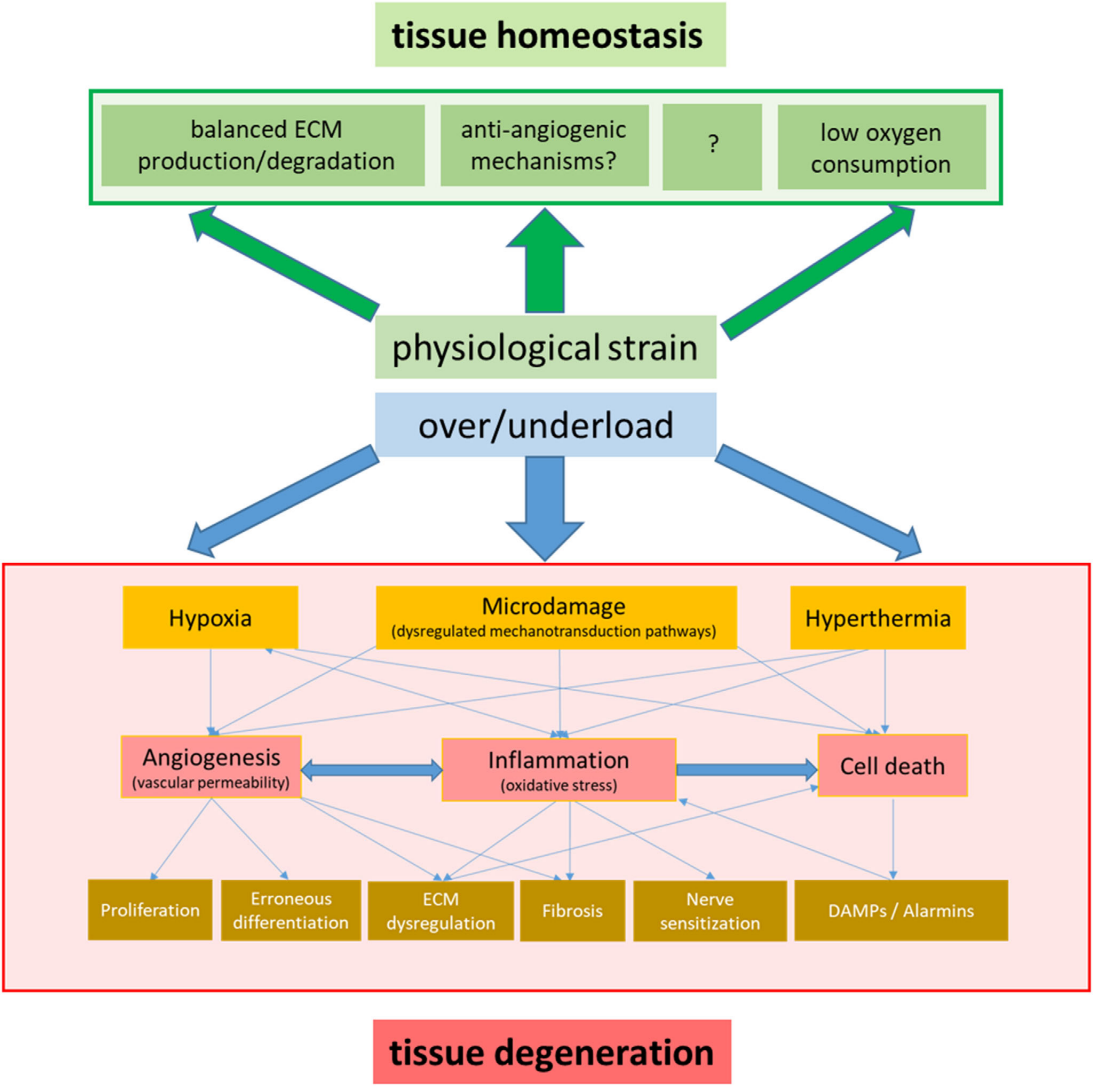
Illustration of the key factors implicated in the development of tendinopathy, highlighting the complex interplay of conditions and mechanisms affecting histopathological alterations seen in tendinopathic tissue.

Summarizing the sections so far presented, **Figure** **12** seeks to integrate the mechanisms underlying tendinopathy so far described in the literature. It displays the intricate interplay between physical/chemical cues and various biological processes involved in the onset and progression of the disease.

### 4.4 Tendinopathic Loop

**Figure 13:**
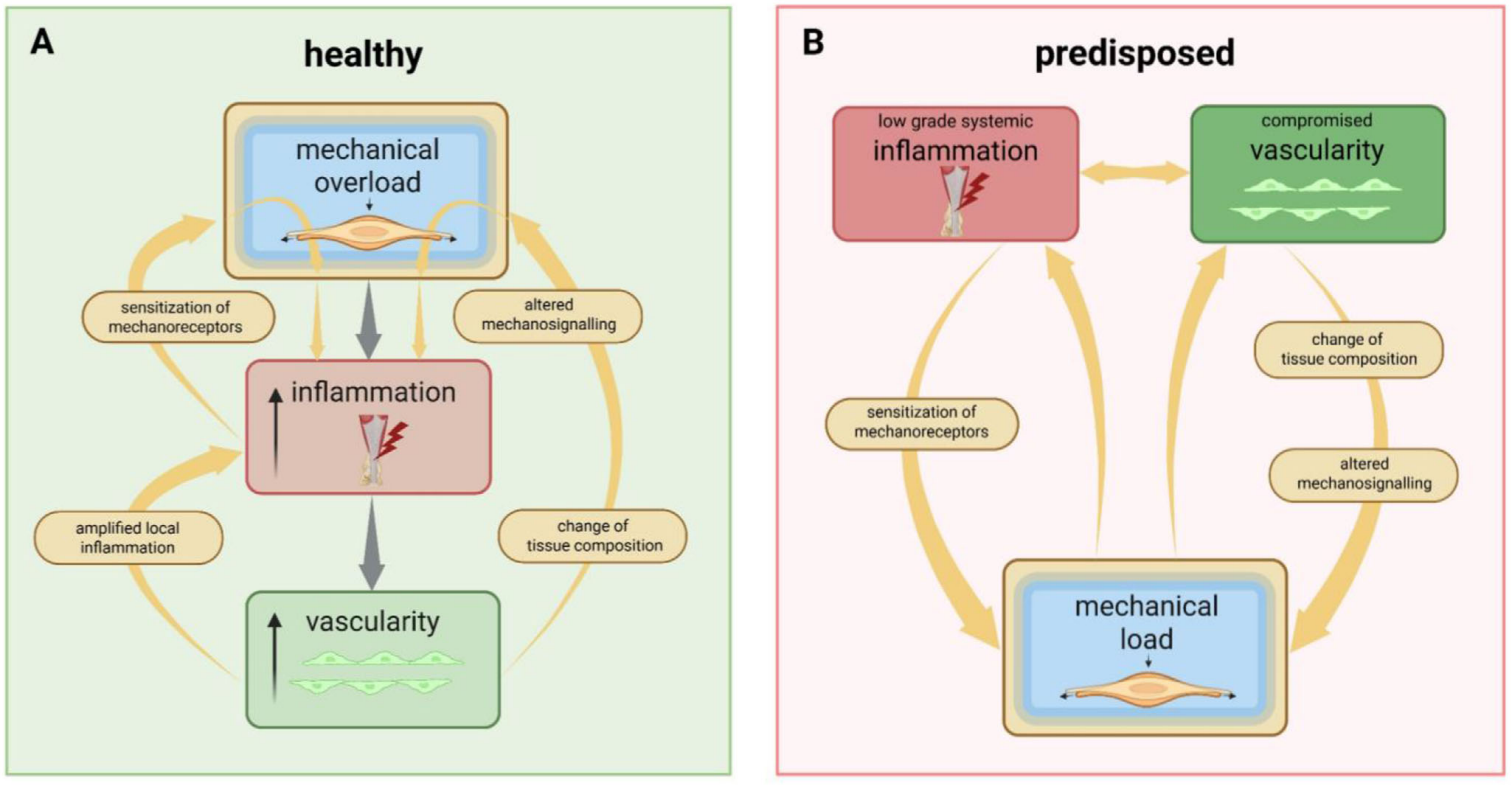
Schematic illustrating the interaction between the three key drivers of tendinopathy. In healthy tendons, repetitive mechanical (over)load initiates the physiological response cascade. In contrast, in tendons of predisposed individuals, inflammation and vascular changes mutually reinforce each other. This results in inflammation‐induced sensitization of mechanoreceptors, changes in tissue composition, and impaired mechanosignaling, all of which feedback to alter the perception and response to mechanical load. Created with BioRender.com.

We propose that the interplay between the three main drivers of tendinopathy differs between healthy tendons and those predisposed by factors such as metabolic disease or the use of medications like fluoroquinolones or statins (**Figure** **13**). In healthy individuals, tendon overloading typically initiates inflammation, which subsequently leads to vascular alterations. In contrast, predisposed tendons exhibit a different baseline state, where low‐grade systemic inflammation and/or vascular changes—due to aging, hypoxia, or drug intake—mutually influence each other, resulting in altered load perception and disrupted mechanosignaling. When this cycle is further driven by repeated overloading, the cumulative release of inflammatory mediators can act synergistically to intensify inflammation. This, in turn, may further impair vascular integrity, increasing vessel permeability and allowing serum components—normally excluded from the tissue under healthy conditions—to infiltrate the tendon environment. Moreover, the inflammatory environment may lead to the sensitization of mechanoreceptors such as Piezo1 lowering their threshold for activation as has been shown for chondrocytes.^[^ ^97^, ^181^, ^182^ ^]^

## 5 Emerging Concepts and Future Directions

To date, no fully conclusive model of tendinopathy has been proposed that integrates all aspects of the disease. Appropriate conceptual frameworks are likely to continue evolving and, in the future, may also incorporate emerging ideas—some of which will be discussed in the following sections.

### 5.1 Intratendinous Pressure as a Novel Factor

Another aspect that did not gain sufficient attention in the last decades is the role of intratendinous pressure in the onset and the progression of tendinopathy. The pathologic transformation of tendon tissue into fibrocartilage‐like tissue may lead to elevated intratendinous resting and dynamic pressure, primarily due to an overabundance of water‐retaining glycosaminoglycans and proteoglycans. This elevated intratendinous resting pressure may contribute to a hypoxic environment due to mechanically reduced blood flow and thus to the development of leaky (neo) vessels associated with tendon pathology. Also, pain may be attributed to increased resting pressure, as many subtypes of pain receptors are also pressure sensitive.^[^ ^175^ ^]^

Recent work by Pringels et al. has revealed that the resting intratendinous pressure in the healthy Achilles tendon averages ≈43.8 ± 15.2 mmHg, and increases linearly with plantarflexion force, surpassing 100 mmHg during a modest 50 N dorsiflexion load.^[^ ^183^ ^]^ These values are notably higher than those typically seen in other musculoskeletal tissues, suggesting that the Achilles tendon is subject to a uniquely pressurized environment even at rest. The study further demonstrated that as intratendinous pressure rises with dorsiflexion, perfusion pressure falls below zero, indicating a potential compromise in blood supply under relatively low mechanical demands. This inverse relationship suggests that the tendon's vasculature is heavily influenced by mechanical loading, even before reaching high exertion levels.

Additional biomechanical studies demonstrate that intrinsic tendon torsion increases intratendinous pressure exponentially during dorsiflexion, particularly during eccentric contractions—highlighting that anatomical factors like twisting subtendons may exacerbate pressure elevations in certain individuals.^[^ ^175^ ^]^

Taken together, these studies suggest that the Achilles tendon operates in a relatively ischemic microenvironment, even under low‐intensity stress, and mechanical factors can further restrict perfusion. This has important implications for tendon physiology and pathology: chronically elevated intratendinous pressure may contribute to ischemic damage and predispose the tendon to tendinopathy.

### 5.2 Cellular Metabolism as a Potential Target

Metabolic diseases such as insulin resistance, diabetes, and obesity contribute to tendinopathy and impaired tendon healing by altering tenocyte metabolism. These systemic conditions reduce glycolytic efficiency and mitochondrial function, while hyperglycemia and insulin resistance promote the accumulation of advanced glycation end‐products (AGEs) and oxidative stress. This leads to collagen crosslinking, increased matrix stiffness, inflammation, and increased risk of rupture. Despite emerging insights, the metabolic regulation of tendon cells—especially under acute or chronic stress—remains incompletely understood.^[^ ^10^ ^]^

However, metabolomic profiling is beginning to bridge the gap between systemic and cellular metabolism in tendon pathology. It reveals links between energy imbalance, oxidative stress, lipid dysregulation, and amino acid depletion. Tendon cells have a distinct metabolic profile tailored to their mechanical function, predominantly relying on oxidative phosphorylation for homeostasis and repair, glycolysis under anaerobic or pathological conditions, and fatty acid oxidation for sustained function—though the latter becomes dysregulated under stress. Amino acid shortages, particularly of proline and glycine, hinder collagen synthesis, while injured tendons often accumulate lactate, glutamate, and pyruvate.^[^ ^184^, ^185^ ^]^ Metabolic shifts in tendinopathy favor glycolysis, leading to local acidosis due to, e.g., lactate accumulation. Inhibiting lactate production improves tendon structure and biomechanics during healing, indicating that an altered glucose metabolism improves tendon repair. Loss of SCX expression has been linked to increased oxidative phosphorylation and mitochondrial organization, underscoring the role of tendon‐specific transcription factors in metabolic control. Tendon cells of injured human tendons display enhanced glycolytic activity and the capacity for both tenogenic and chondrogenic differentiation.^[^ ^186^ ^]^ Blocking glycolysis with 2‐deoxy‐d‐glucose (2DG) suppresses chondrogenic differentiation and promotes a tenogenic phenotype, suggesting that glucose metabolism directly influences repair outcomes.^[^ ^186^ ^]^ Inflammation also plays a metabolic role. IL‐1β increases lactate production, reduces collagen synthesis, and downregulates tendon‐specific markers. While hypoxia (1–5% O_2_) enhances proliferation and stemness by promoting glycolysis and limiting mitochondrial output, severe hypoxia or anoxia triggers apoptosis. Reactive oxygen species (ROS) have dual roles—regulating inflammation and ECM remodeling under physiological conditions but driving pathology when unchecked. Antioxidants like vitamin C can reverse ROS‐induced damage.^[^ ^187^, ^188^ ^]^

Fatty acid oxidation has emerged as a key pathway in tendon repair. AMP‐activated protein kinase (AMPK), a master regulator of energy homeostasis, is highly expressed in regenerative tendons. Its inhibition impairs healing and reduces expression of CPT1A, a mitochondrial enzyme critical for stem/progenitor cell proliferation and tenogenic differentiation.^[^ ^189^ ^]^ In tendinopathic tissues, AMPK is downregulated, contributing to ECM degeneration, mechanical weakness, and cellular senescence.^[^ ^190^ ^]^

Accumulating evidence suggests important pathological roles of dysregulated mechanotransduction in metabolic diseases, including nonalcoholic fatty liver disease (NAFLD) and type 2 diabetes mellitus (T2DM.)^[^ ^191^ ^]^ Mechanotransduction is emerging to regulate a diversity of cell metabolic proceedings, including glucose uptake, glycolysis, the transition from glycolysis to oxidative phosphorylation system (OXPHOS), metabolic redox homeostasis, lipid metabolism and others.^[^ ^192^ ^]^ Particularly YAP/TAZ serve as an essential node in modifying the metabolic availability of cells under mechanical stimuli.^[^ ^192^ ^]^ Intriguingly, mechanical stimuli can also change the morphologies and structures of the golgi apparatus and mitochondria by regulating cytoskeletal dynamics and forces, offering attractive links to cellular metabolic states.^[^ ^191^ ^]^

Though key mechanisms remain unresolved, growing evidence suggests that targeting tendon metabolism could enhance resilience and healing. Strategies to support metabolic homeostasis, regulate oxidative stress, and preserve collagen synthesis hold promise for preventing and treating tendon disorders.

### 5.3 Treatment Options

The diagnosis of tendinopathy is primarily based on clinical symptoms, including pain, swelling, and potentially the presence of neovascularization. However, it remains uncertain whether tendinopathy constitutes a single, uniform disease entity, as there is currently no commonly accepted biomarker described. It is very likely that multiple endotypes of tendinopathy exist, differing in etiology, pathophysiology, and progression. This heterogeneity complicates both research efforts and the development of targeted treatments. Due to the limited understanding of the underlying pathophysiological mechanisms, treatment options for tendinopathies remain restricted. Currently, clinicians can choose between conservative management—typically involving medication and physical therapy to alleviate pain and inflammation—or surgical intervention for tendon debridement.

A recent comprehensive analysis of systematic reviews, limited to randomized controlled trials, assessed the effectiveness of various conservative treatments for tendinopathies. Among physiotherapy‐based interventions—including extracorporeal shock wave therapy, ultrasound, laser therapy, needling, and eccentric exercise—eccentric exercise emerged as the most effective approach, a finding further supported by additional studies.^[^ ^193^, ^194^ ^]^

By targeting metabolic pathways, it might be possible to reprogram the tendon cell function, dampen or even prevent tendon degeneration, reduce inflammation or enhance tendon healing, offering new therapeutic strategies beyond mechanical or surgical interventions. Such new treatment options could involve promoting mitochondrial biogenesis and oxidative phosphorylation using agents like AMPK activators (e.g., metformin or AICAR) or PGC‐1α agonists.^[^ ^195^ ^]^ PGC‐1α agonists show promising effects in treating fibrosis, as PGC‐1α activation has been shown to counteract metabolic shifts driving pro‐fibrotic responses, particularly in tissues like the lung, liver, kidney, and heart.^[^ ^196^, ^197^ ^]^ Nutrient‐sensing pathways such as mTOR and sirtuins may also be targeted to restore cellular homeostasis. Additionally, interventions like dietary factors might positively impact tendon metabolism enhancing metabolic flexibility and reducing low‐grade inflammation.^[^ ^198^, ^199^ ^]^ Although further research is needed, these approaches could not only help manage tendinopathy symptoms but also reprogram tendon cell metabolism to promote functional tissue repair.

## 6 Current Challenges and Existing Research Gaps

The cascade of overuse, inflammation, matrix damage and hypervascularity is complex, and so far it remains to be proven which is the most dominant trigger of tendinopathy in humans. As tendinopathy is a multifactorial disease, with several potential trigger points, most likely there is no single cause for the initiation and progression of tendon disease.

For studying the events particularly in early tendinopathy, which is most likely subclinical, suitable animal models are urgently needed. The currently available models mimic symptoms of tendinopathy, such as matrix disruption in case of collagenase injection, or tendon overuse, e.g., by treadmill running. Although both models are artificial, the mechanical overuse‐model very likely is better able to replicate a naturally occurring tendinopathy and is so far the best tendinopathy model we have at hand.

Moreover, it needs to be distinguished if “overload” is referred to as an impact acutely causing macro‐ or microdamage to the tendon matrix (even as a singular event), or if overloading, either due to duration, frequency or amplitude, stimulates tendon cells to weaken their matrix, produce inflammatory factors and attract new blood vessels. Both of these overload situations definitely occur in human pathology, but the underlying mechanisms much likely differ from each other. Therefore, there is no “one‐size‐fits‐all” tendinopathy animal model.

In addition to appropriate animal models, several other aspects of tendon biology must be addressed. Although challenging, understanding these factors is an essential prerequisite for successful tendon regeneration. These aspects are essential for establishing physiologically relevant conditions for ex vivo and in vitro experiments including appropriate oxygen tension and applied mechanical strain.

In most tissues, hypoxia following injury is a key driver of all subsequent reparative or scar forming processes. Healthy tendon tissue being sparsely vascularized is likely to be confronted with low oxygen tension. However, to our knowledge, this oxygen tension has not been extensively examined so far, thus more information about the actual oxygen content in tendons in vivo would be urgently needed. Yet, cellular oxygen stress is always a result of oxygen demand exceeding availability. Therefore, the role of metabolic changes in tendon cells under pathological conditions, particularly in relation to oxygen availability, should be addressed infuture studies.

Regarding the “Angiogenic Landscape” of healthy tendon, the balance of pro‐ and antiangiogenic mechanisms, further research is warranted to improve our understanding of the mechanisms preventing vascular ingrowth. In other sparsely vascularized tissues such as the cornea, a plethora of factors have been described, preventing angiogenesis such a soluble VEGF receptors, which have not been studied in tendon so far.^[^ ^79^ ^]^

Along the same lines, the question of vascular permeability in tendons needs to be addressed. Although neovessels are known to be leaky, it has yet to be demonstrated in vivo whether blood vessel permeability increases under pathological conditions in the same way as described for other tissues, such as the brain.

## 7 Summary and Conclusion

Summarizing, tendinopathy is a highly complex and multifaceted disease which is difficult to target due to a still fragmentary understanding of its etiology. Since 95% of all tendon ruptures are preceded by degenerative alterations of the tissue, the need to better define the underlying pathomechanisms in order to take preventive measures becomes evident.

**Figure 14:**
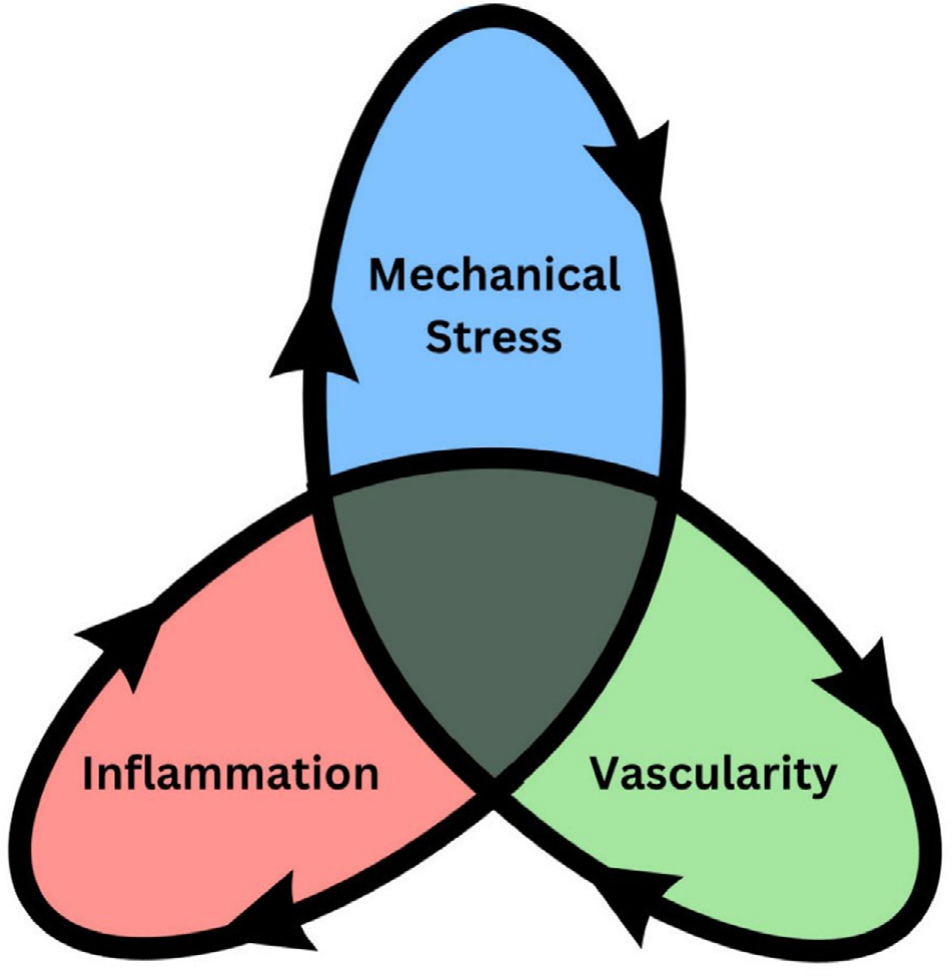
If left unresolved, tendinopathy can progress in a vicious cycle—an escalating, self‐perpetuating loop fueled by mechanical stress, inflammation, and altered vascularity, each amplifying the others. Interrupting this cycle at any point is likely to slow its progression, which is why a combination of therapies that independently target these key drivers currently appears to be the most promising treatment approach.

As displayed in **Figure** **14** we understand the development of tendinopathy as an intricate interplay between three main drivers, namely mechanical stress, inflammation and vascularity. All three factors influence and work back on each other, their interdependence being fueled by additional extrinsic as well as intrinsic parameters such as, e.g., weight, genetic factors and certain drugs. Finally, this synergistic effect inducing downstream effects including apoptosis, fibrosis, proliferation, neo‐vascularization and neo‐innervation, respectively, manifests in a disease commonly referred to as tendinopathy. We picture tendinopathy as a dynamic process that can be initiated at any point by surpassing the physiological threshold of any of the three main drivers, and once returning to tissue homeostasis through resolving mechanisms fails, the “circulus vitiosus” keeps going leading up to a chronic tendinopathic state.

As for now, although much desired and searched for, we unfortunately do not have the one therapy or cure at hand which could be universally used to treat tendinopathy. Due to the still not fully unravelled interrelationship of the main drivers presented in this review, it is more than questionable whether we will be able to find this “main switch” which could be precisely targeted. In the future, targeting metabolic processes may prove to be a central therapeutic strategy—provided their role in tendinopathy is better understood. Until more targeted therapies are available, the complexity of the disease—both in its etiology and progression—requires us to rely on a combination of approaches, including rest, anti‐inflammatory treatment, and potentially anti‐angiogenic therapies. These strategies have shown benefits by addressing different underlying causative mechanisms.

## Conflict of Interest

The authors declare no conflict of interest.
